# Endotypes of severe neutrophilic and eosinophilic asthma from multi‐omics integration of U‐BIOPRED sputum samples

**DOI:** 10.1002/ctm2.1771

**Published:** 2024-07-28

**Authors:** Nazanin Zounemat Kermani, Chuan‐Xing Li, Ali Versi, Yusef Badi, Kai Sun, Mahmoud I Abdel‐Aziz, Martina Bonatti, Anke‐Hilse Maitland‐van der Zee, Ratko Djukanovic, Åsa Wheelock, Sven‐Erik Dahlen, Peter Howarth, Yike Guo, Kian Fan Chung, Ian M. Adcock, U‐BIOPRED Project Team

**Affiliations:** ^1^ National Heart and Lung Institute Imperial College London London UK; ^2^ Data Science Institute Imperial College London London UK; ^3^ Respiratory Medicine Unit Department of Medicine & Centre for Molecular Medicine Karolinska Institutet Stockholm Sweden; ^4^ Department of Pulmonology Amsterdam UMC University of Amsterdam Amsterdam The Netherlands; ^5^ NIHR Southampton Respiratory Biomedical Research Unit and Clinical and Experimental Sciences Southampton UK; ^6^ Institute of Environmental Medicine Centre for Allergy Research Karolinska Institute Stockholm Sweden

**Keywords:** asthma endotype, consensus clustering, eosinophilic inflammation, gene set variation analysis, neutrophilic inflammation, pathogenic bacteria, severe asthma, similarity network fusion

## Abstract

**Background:**

Clustering approaches using single *omics* platforms are increasingly used to characterise molecular phenotypes of eosinophilic and neutrophilic asthma. Effective integration of multi‐omics platforms should lead towards greater refinement of asthma endotypes across molecular dimensions and indicate key targets for intervention or biomarker development.

**Objectives:**

To determine whether multi‐omics integration of sputum leads to improved granularity of the molecular classification of severe asthma.

**Methods:**

We analyzed six ‐*omics* data blocks–microarray transcriptomics, gene set variation analysis of microarray transcriptomics, SomaSCAN proteomics assay, shotgun proteomics, 16S microbiome sequencing, and shotgun metagenomic sequencing–from induced sputum samples of 57 severe asthma patients, 15 mild‐moderate asthma patients, and 13 healthy volunteers in the U‐BIOPRED European cohort. We used Monti consensus clustering algorithm for aggregation of clustering results and Similarity Network Fusion to integrate the 6 multi‐omics datasets of the 72 asthmatics.

**Results:**

Five stable omics‐associated clusters were identified (OACs). OAC1 had the best lung function with the least number of severe asthmatics with sputum paucigranulocytic inflammation. OAC5 also had fewer severe asthma patients but the highest incidence of atopy and allergic rhinitis, with paucigranulocytic inflammation. OAC3 comprised only severe asthmatics with the highest sputum eosinophilia. OAC2 had the highest sputum neutrophilia followed by OAC4 with both clusters consisting of mostly severe asthma but with more ex/current smokers in OAC4. Compared to OAC4, there was higher incidence of nasal polyps, allergic rhinitis, and eczema in OAC2. OAC2 had microbial dysbiosis with abundant *Moraxella catarrhalis* and *Haemophilus influenzae*. OAC4 was associated with pathways linked to IL‐22 cytokine activation, with the prediction of therapeutic response to anti‐IL22 antibody therapy.

**Conclusion:**

Multi‐omics analysis of sputum in asthma has defined with greater granularity the asthma endotypes linked to neutrophilic and eosinophilic inflammation. Modelling diverse types of high‐dimensional interactions will contribute to a more comprehensive understanding of complex endotypes.

**Key Points:**

- Unsupervised clustering on sputum multi‐omics of asthma subjects identified 3 out of 5 clusters with predominantly severe asthma.
- One severe asthma cluster was linked to type 2 inflammation and sputum eosinophilia while the other 2 clusters to sputum neutrophilia.
- One severe neutrophilic asthma cluster was linked to *Moraxella catarrhalis* and to a lesser extent *Haemophilus influenzae* while the second cluster to activation of IL‐22.

## Graphical abstract

- Multi‐omics analysis of asthma sputum identified five omics‐associated clusters (OAC1‐5).
- OAC2–OAC4 consist of patients with severe asthma while OAC1 and OAC5 mainly contain mild‐to‐moderate asthmatics.
- OAC3 represents a Th2 eosinophilic cluster.
- OAC2 and OAC4 are distinct severe mixed and neutrophilic asthma clusters with different neutrophil subtypes and activated pathways.

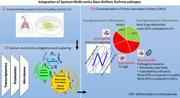

## 1 INTRODUCTION

Asthma is a heterogeneous chronic inflammatory disease of the airways with many clinical phenotypes^1^ including severity based on the level of treatment needed to control symptoms.^2^ Patients with type 2 (T2) high inflammation have been defined as those with frequent exacerbations, high blood or sputum eosinophils and raised levels of fractional exhaled nitric oxide (FeNO).^3^ These patients are more likely to respond to corticosteroid therapy and to biologics targeted against the cytokines of the T2 pathway, such as anti‐IL5 and anti‐IL5R antibodies or the anti‐IL4R antibody that blocks the effect of IL4 and IL13.^4^, ^5^ Another inflammatory severe asthma phenotype characterised by sputum neutrophilia characterises neutrophilic and mixed granulocytic asthma that has a poor response to corticosteroid therapy but so far, no targeted antibody therapies are currently available for this non‐T2 inflammatory phenotype.^6^, ^7^, ^8^

Clustering of patients with severe asthma using either transcriptomic or proteomic analytical approaches has helped to define the molecular pathways underlying these different inflammatory phenotypes using sputum samples, which has been used to define the granulocytic inflammation.^9^, ^10^ Three transcriptome‐associated clusters were described including one with sputum eosinophilia characterised by immune receptors IL33R, CCR3 and TSLPR and high enrichment of gene signatures for interleukin‐13/T‐helper cell type 2 (Th2) and another one with sputum neutrophilia characterised by interferon, tumour necrosis factor‐α and inflammasome‐associated genes.^9^ Using proteomic data analysis, ten clusters were described with the definition of 3 highly eosinophilic, 3 highly neutrophilic, and 2 highly atopic with relatively low granulocytic inflammation.^10^ In addition, sputum analysis has yielded two microbial clusters with one having elevated levels of the pathogenic *Haemophilus influenzae* and *Moraxella catarrhalis*.^11^

Systems biology defined as ‘an approach to understanding living systems that focuses on modelling diverse types of high‐dimensional interactions to develop a more comprehensive understanding of complex phenotypes manifested by the system’ remains a key strategy for improving the outputs from the high‐throughput data.^12^ Although these single *omics* studies of severe asthma have yielded new molecular clusters, we hypothesised that integrating the several *omics* platforms of data will likely enable more holistic and granular models that reflects the complex inter‐molecular interactions of different pathways, and proteins, transcripts and of microbiome and the perturbations in asthma. Thus, combining multi‐omics datasets will generate clear subsets of severe asthma that are associated with different pathways and pathogenic organisms. We therefore integrated sputum transcriptomics (microarray and pathway enrichment scores), proteomics (shotgun and SomaLogic) together with 16S and metagenomics using the consensus clustering algorithm and similarity network fusion to generate omics‐associated clusters (OACs). Five endotypes were identified; three were severe asthma endotypes: two were associated with sputum neutrophilia with one linked predominantly with *Moraxella catarrhalis* and to a lesser extent *Haemophillus influenzae*, while the other with a drug response signature to anti‐IL‐22 antibody treatment, and the third one with sputum eosinophilia.

## 2 METHODS

### 2.1 Participants and data collection

**FIGURE 1:**
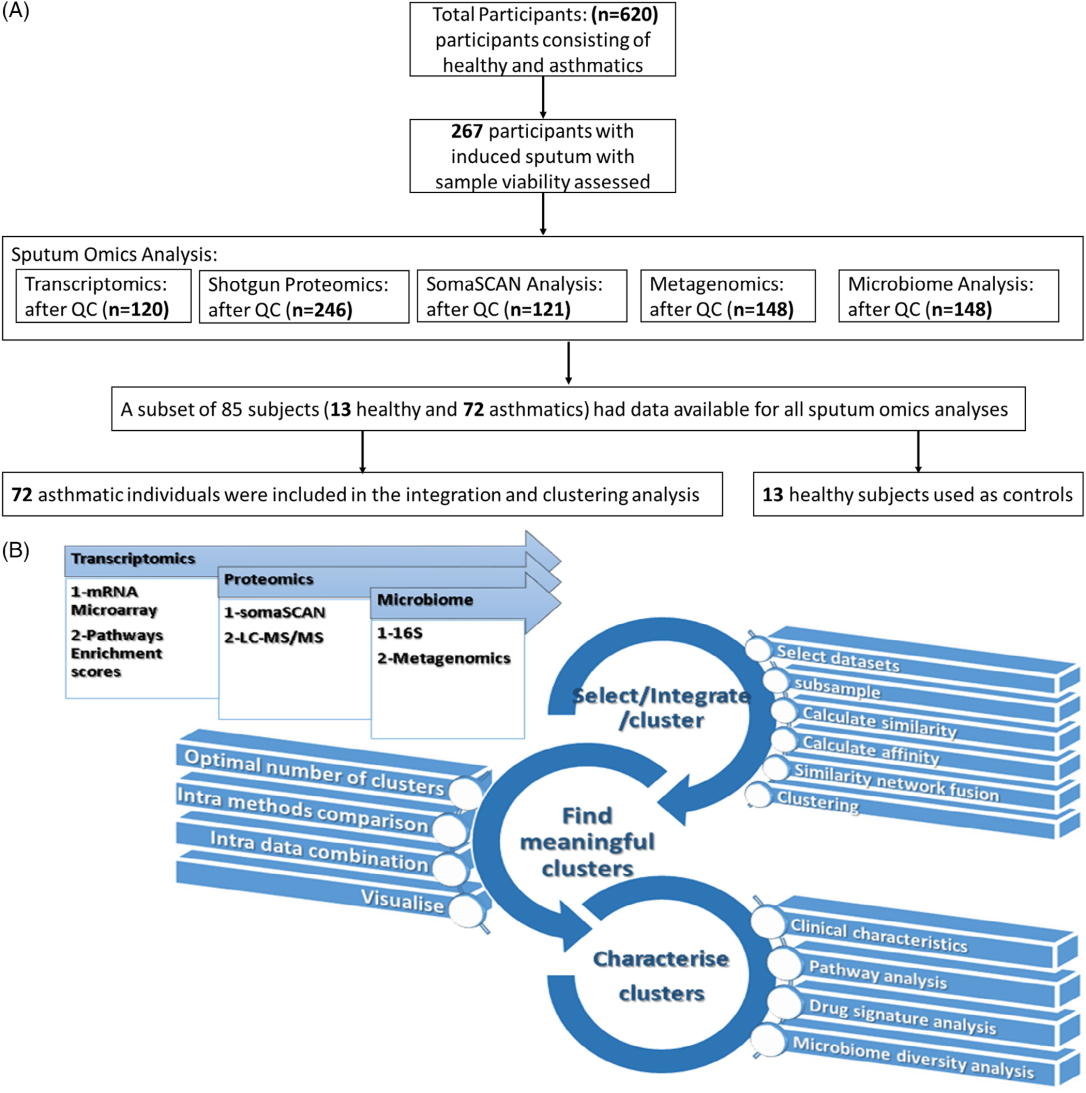
Machine learning workflow. (A) CONSORT flow chart reporting the patients and controls we studied. (B) Three types of data were the starting point of the workflow, that is, transcriptomics, proteomics and microbiome date. Each of these three data types included 2 data matrices either derived by incorporating available knowledge or from various bioanalytical platforms, for example, proteomics data from LC‐MS/MS and somaSCAN, microbiome from 16S and Metagenomics platforms. The workflow consists of three main multi‐faceted compartments: (1) select/integrate/cluster, (2) find meaningful clusters, (3) characterise clusters. The first compartment is about running a data integration and clustering algorithm on different combination of data and generating clusters. The second compartment includes multiple steps to calculate, compare and visualise various groupings generated by the first compartment. The goal of this step is to aid decision making about the optimal number of clusters. Finally, the third compartment characterises the only clustering result that is deemed to be most suitable and stable through the second compartment.

The European‐wide U‐BIOPRED (Unbiased BIOmarkers in PREDiction of respiratory disease outcomes) cohort consisted of 4 groups of participants: severe nonsmoking asthma, ever smokers with severe asthma, mild/moderate nonsmoking asthmatics (MMA) and nonsmoking healthy volunteers (HV, *n* = 101).^13^ Out of this, a subset of 85 subjects (13 healthy and 15 mild‐to‐moderate, 37 severe nonsmokers and 20 severe ever smokers asthmatics), who produced sputum cells for RNA extraction (Table S1) and expression profiling undertaken as previously described,^9^, ^14^ were included in the current analysis. A CONSORT diagram is provided in Figure 1A. The 72 subjects included had similar characteristics to the remainder of the cohort (Table S1). In addition, shotgun and SomaScan proteomics^10^ and 16S sequencing and metagenomics^15^ were also undertaken in sputum samples, as previously described. All U‐BIOPRED participants gave signed informed consent to participate in the study, which was approved by the local Ethics Committee of each country.

Data were downloaded from the tranSMART database on 2 February 2020. Seventy‐two asthmatics were aligned across omics datasets (Table S2), with the data from healthy volunteers excluded from the datasets but acting as a control group. All datasets were checked for features with zero variance (ZV) with 16S and metagenomics datasets having 71 and 14 features with ZV, respectively. We derived enrichment scores of 1471 pathways from a curated pathways database (GeneGO, Thomson Reuters, http://portal.genego.com) using the transcriptomics matrix and gene set variation analysis (GSVA).^17^ By adding pathways’ information as a data‐block, we capture more robust and interpretable patterns in the data that can be captured by the transcriptomics data alone, thereby reducing the impact of noisy or irrelevant features of the transcriptomics matrix that could exacerbate the curse of dimensionality Figure 1B.

### 2.2 Monti consensus clustering and similarity network fusion (SNF)

We employed the Monti consensus clustering algorithm to aggregate clustering results from multiple subsamples of the data.^19^ This approach allowed us to identify groupings of the data that were robust and less sensitive to the exclusion of individual subjects. The consensus clustering was conducted using the ConsensusClusterPlus package in R (version 1.48.0).

Additionally, we integrated Similarity Network Fusion (SNF)^16^ into the consensus clustering process. SNF enables the integration of multiple datasets by computing affinity matrices from distance matrices, which capture pairwise similarities between samples in each dataset. These affinity matrices are then combined to construct a consensus network that integrates information from all datasets, enabling the discovery of underlying relationships and patterns across heterogeneous data sources. For transcriptomics and proteomics datasets, we used the Euclidean distance measure, while for 16S and metagenomics datasets, we utilised the ‘m’ and ‘−2′ beta‐diversity measures, respectively, as provided by the ‘vegan’ R package.

We calculated affinity matrices for each dataset and set the parameters for SNF to include 20 neighbours with an alpha value of 0.5. These affinity matrices were then integrated using the SNF algorithm, with parameters set to 20 neighbours and 50 iterations. We conducted two separate runs of SNF‐based clustering using finite Gaussian mixture clustering (GMM)^20^ and spectral clustering methods.^21^

While spectral clustering is the preferred method for SNF, our previous observations suggested that applying finite GMM to SNF‐derived vectors produced similar but more stable clusters.^22^ We determined the optimal number of clusters by comparing variations in clustering results across different combinations of the data.

### 2.3 Determining the optimal number of clusters

To identify the optimal cluster number, we evaluated clustering results using both GMM and spectral methods across seven possible combinations of the data (Table S3). We utilised the Adjusted Rand Index (ARI) to measure the similarity between clustering results.^23^ A higher ARI value indicates greater similarity between clustering methods.

We visualised the results by plotting the cluster number against the ARI value and selecting the clustering with a relatively high ARI between the two methods and minimal variation across data combinations. The optimal cluster number was determined by identifying a point where the relative change in the cumulative distribution function (CDF) became minimal and stable, with cluster number *k* = 5 being selected as optimal based on this criterion as demonstrated under ‘find meaningful clusters’ in Figures 1B and S2.

### 2.4 Clinical, molecular and pathway characteristics of clusters

We analysed the clusters according to (i) clinical/demographical/haematology/quality of life questionnaires/sputum cell counts, (ii) pathway analysis, (iii) drug signature analysis and (iv) microbiome diversity. For the clinical features, a chi‐squared test was used for computing the *p* value of categorical variables while the normality of numerical variables was examined by the Shapiro–Wilk test. Mann–Whitney *U* test was used for nonnormally distributed numerical variables and a pairwise Student's *t*‐test for normally distributed numerical variables. The significance level used for hypothesis testing was specified at α = .05, with multiple testing correction applied using the False‐Discovery Rate (FDR) method.

GSVA was applied to each of these datasets to calculate the enrichment scores (ES) for the 1471 pathways that were recorded in the curated pathways database (GeneGO, Thomson Reuters, http://portal.genego.com) based on the transcriptomics, SomaScan and shotgun proteomics datasets.^17^ The minimum overlap between datasets and pathways were set to 3 to ensure that the ES were calculated only for pathways with at least 3 genes in common. A word cloud visualisation was used to visualise genes belonging to the 31 common pathways, which were frequently differentiated between clusters. We hypothesised that each cluster would deviate from the healthy population and other clusters in a unique manner to generate heterogeneous clinical and molecular characteristics. GSVA was also used to determine the neutrophilic activation mechanisms utilising enrichment of previously defined gene signatures and neutrophil subtypes^18^ in the OAC2, OAC4 and healthy controls. When a cluster comprised both severe asthma and mild‐to‐moderate asthma cases (e.g. OAC1 and OAC5) and contained more than five mild‐to‐moderate asthmatics (e.g. OAC1), we conducted differential gene/protein analysis to elucidate the pathogenic mechanisms distinguishing severe from nonsevere asthma.

We next analysed the 5 OACs according to ActivePathways.^16^ ActivePathways integrates statistical information from multiple omics datasets using a Brown's method to prioritise biologically relevant pathways. It then applies a hypergeometric test to identify overrepresented pathways, providing a robust approach for pathway analysis in the presence of multiple omics datasets. We used molecular pathways of the Reactome database (version 82) and EnrichmentMap app of Cytoscape for network visualisation. We introduced a new concept for finding subjects that are more likely to respond to a drug, called the ‘N’ method. We illustrated this method using ‘Fezakinumab’. It is an anti‐IL22 antibody used to block the effect of the IL‐22 cytokine and has been approved for the treatment of atopic dermatitis. First, we derived disease and drug response gene signatures from recent publications^27^ and used GSVA to study the distribution of the drug/disease signature ES across the clusters. We hypothesised that if a cluster was up‐ or downregulated for the disease signature and was in the right direction for the corresponding drug signature(s), that group might respond to this drug or antibody.

16S and metagenomics data were used to calculate Shannon α‐diversity across the clusters. The differential bacterial abundance between clusters was computed using edgeR (R package edgeR version 3.26.8) after geometric mean of pairwise ratios (GMPR) normalisation.^28^ For intracluster comparison and comparison with healthy population, we used the nonparametric Kruskal–Wallis Rank Sum test. To minimise the number of false positives, for each group comparison, we collated the *p* values derived from all datasets and adjusted the *p* values using Benjamini‐Hochberg FDR method. We plotted the histogram of the *p* values and a heatmap for each dataset was used to visualise the overall between‐group differences (Figure S1).

### 2.5 Network visualisation

Datasets undergo feature selection to identify key features distinguishing OAC within each omics domain. Each dataset undergoes an application of the Prediction Analysis for Microarrays (PAMR) framework.^17^ For each OAC, a structure is formed, integrating gene/protein/microbiome IDs and class labels. The PAMR model is trained with a positive sign contrast to discern upregulated genes specific to the OAC subtype. A 10‐fold cross‐validation is performed to assess the predictive accuracy of the model, with the minimum cross‐validated error reported as a metric of model performance.

Subsequently, signature matrices are constructed for each dataset, capturing distinctive molecular patterns associated with OACs. These matrices are then amalgamated across datasets and underwent clustering for community detection, allowing for a high level visualisation of holistic differences of OAC‐related alterations at the transcriptomics, proteomic and microbiomic levels.^18^

The ARENA3D^19^ tool was employed to create an interactive three‐dimensional representation of the correlation networks. This tool allows for a dynamic exploration of the networks, facilitating the identification of densely connected clusters or modules and highlighting potential hub signatures that exhibit strong correlations across omics layers.

## 3 RESULTS

### 3.1 Clustering and optimisation of cluster number

The optimal number of clusters was determined as *k* = 5. The 5 clusters exhibit a relatively small increase of the relative change in the CDF at the *k* + 1 cluster analysis. Changes were minimal after *k* = 5 and had the highest ARI after that for three clusters, but *k* = 5 had the smallest variance. This demonstrates that 5 clusters are less sensitive to clustering methods and combination of the data. Thus, clustering using consensus clustering and SNF identified 5 stable omics‐associated clusters (OAC1‐5) (Figure S2).

### 3.2 Clinical and physiological characteristics of OAC1–5

The 5 clusters generated according to the multi‐omics integration of data had distinct features that generally mapped to known clinical subtypes of asthma (Table 1). Thus, OAC1 (*n* = 20) had the lowest percentage of severe asthmatics (50%) and oral corticosteroid use (10%), compared to the 4 other clusters. OAC1 also has the highest FEV_1_ % predicted compared to all other OACs except for OAC5 where the difference did not reach significance. OAC3 (*n* = 18) patients are all severe asthmatics with the highest level of sputum eosinophils (35.5%, Table 1) and highest oral corticosteroid use (56%). Sputum eosinophil levels were significantly higher in OAC3 subjects compared to patients in OAC1, OAC2 and OAC4 (Table 1).

**TABLE 1:** Clinical features of the 5 sputum omics‐associated clusters (OACs).

	OAC 1	OAC2	OAC3	OAC4	OAC 5	Group comparison (*p* values)									
Subjects	20	12	18	12	10	1 vs. 2	1 vs. 3	1 vs. 4	1 vs. 5	2 vs. 3	2 vs. 4	2 vs. 5	3 vs. 4	3 vs. 5	4 vs. 5
**Age (years)**	47 (31.8–53.5)	54.5 (41.2–58.8)	52.5 (46.2–58)	54 (49.5–63.5)	55 (46.2–63)	NS	NS	NS	NS	NS	NS	NS	NS	NS	NS
**Age of onset (years)**	16 (6–40)	20.5 (14.8–44.5)	37.5 (7.5–47.5)	27 (24.5–46)	18 (6.2–37.8)	NS	NS	NS	NS	NS	NS	NS	NS	NS	NS
**Female**	11 (55%)	9 (75%)	8 (44%)	8 (67%)	4 (40%)	**	NS	NS	NS	**	NS	**	**	NS	**
**BMI**	25.1 (23–32.1)	25.5 (23.6–31.3)	25.8 (24.4–29.6)	31.2 (27.8–32.8)	27.5 (23.2–31.3)	NS	NS	NS	NS	NS	NS	NS	NS	NS	NS
**Smoker (current)**	2 (10%)	0 (0%)	2 (11.8%)	2 (16.7%)	0 (0%)	NS	NS	**	NS	NS	**	NS	**	NS	**
**Nasal polyps (yes)**	3 (15%)	5 (50%)	8 (47%)	3 (25%)	3 (30%)	**	**	NS	*	NS	**	*	**	*	NS
**Allergic rhinitis**	9 (47%)	7 (70%)	5 (36%)	1 (9%)	7 (88%)	**	NS	***	***	***	***	**	***	***	***
**Eczema**	6 (32%)	6 (55%)	4 (24%)	3 (25%)	2 (22%)	**	NS	NS	NS	**	**	**	NS	NS	NS
**Severe asthma**	10 (50%)	11 (92%)	18 (100%)	12 (100%)	6 (60%)	***	***	***	NS	*	**	***	NS	***	***
**Oral corticosteroid use**	2 (10%)	5 (42%)	10 (56%)	4 (36%)	1 (10%)	***	***	**	NS	NS	NS	***	**	***	***
**Atopy (+)**	16 (80%)	9 (75%)	14 (78%)	6 (60%)	8 (80%)	NS	NS	*	NS	NS	NS	NS	*	NS	*
**Exacerbations last year**	1 (0–2.2)	2 (0–2.2)	2 (0–3.8)	2 (0–2.5)	1 (0–2.8)	**	**	NS	NS	NS	NS	NS	NS	NS	NS
**FEV_1_ (% pred)**	86.9 (73–94.4)	57.5 (39.3–76.4)	58.1 (48.7–78.4)	58.3 (44.2–70)	69.6 (60.2–85.5)	*	**	***	NS	NS	NS	NS	NS	NS	NS
**Total IgE (IU/mL)**	95.7 (54.8–168.8)	159 (44.4–367.2)	203 (68.8–370.5)	52 (33–97.5)	86 (34–551)	NS	NS	NS	NS	NS	NS	NS	NS	NS	NS
**Blood leukocytes**	6 (5.2–7.3)	9.5 (7.9–10.9)	8.7 (6.1–10.5)	7.7 (6.3–9.8)	6.9 (6.2–8.3)	*	*	NS	NS	NS	NS	NS	NS	NS	NS
**Blood eosinophils**	0.2 (0.1–0.2)	0.3 (0.25–0.4)	0.4 (0.12–0.5)	0.2 (0.18–0.3)	0.3 (0.19–0.5)	NS	NS	NS	NS	NS	NS	NS	NS	NS	NS
**Blood neutrophils**	3.3 (2.9–4.5)	6.2 (4.6–7.2)	4.6 (3.3–7)	4.4 (3.8–6.6)	4.1 (3.9–4.5)	**	NS	NS	NS	NS	NS	NS	NS	NS	NS
**Sputum eosinophils (%)**	0.5 (0–1.7)	2 (0.19–9.9)	35.5 (11.6–51.4)	4.1 (0.14–7.6)	0.9 (0.18–11.3)	NS	***	NS	NS	*	NS	NS	*	NS	NS
**Sputum neutrophils (%)**	37.5 (29.6–50.7)	91.3 (83.4–93.8)	33.3 (25–60.3)	65.4 (54.1–73.2)	39.8 (20.3–61.9)	****	NS	**	NS	***	**	**	*	NS	NS
**Sputum macrophages (%)**	58.9 (46.8–65.1)	4.4 (3.4–8)	20.5 (14.8–26)	27.5 (21.4–33.9)	38.3 (29.9–65.9)	****	***	***	NS	***	***	***	NS	*	NS
**FeNO (ppb)**	28 (20–49.8)	26 (16.2–45)	41.5 (22.4–68)	31 (10–45.5)	22 (18–29)	NS	NS	NS	NS	NS	NS	NS	NS	NS	NS
**Serum periostin (ng/mL)**	41.8 (32.7–53.4)	49.9 (46.8–61.1)	52.8 (47.4–66.1)	43.3 (36.6–48.86)	44 (38.7–47.7)	NS	NS	NS	NS	NS	NS	NS	NS	NS	NS
**CRP (mg/L)**	0.8 (0.3–2)	3.9 (2.2–14.2)	1.1 (0.8–2.4)	2.5 (1–4.5)	1 (0.6–3.1)	NS	NS	NS	NS	NS	NS	NS	NS	NS	NS
**History pneumonia (+)**	2 (10%)	1 (8%)	2 (11%)	1 (10%)	3 (30%)	NS	NS	NS	**	NS	NS	**	NS	**	**

*Note*: Variables are described as median (interquartile range 25%−75%) or *n* (%). *p* Value: χ^2^ test was used for categorical variables. Normality of numerical variables was examined by the Shapiro–Wilk test. Kruskal–Wallis test was used for nonnormally distributed numerical variables and pairwise Student's *t*‐test for normally distributed numerical variables. Statistical significance: *p < .05; **p < .01; ***p < .001; ****p < .0001; otherwise nonsignificant (*p* > .05).

Abbreviations: BMI, body mass index; CRP, C‐reactive protein; FEV1, forced expiratory volume in 1 s; FeNO, level of nitric oxide in exhaled breath; ns, not significant; ppb, parts per billion.

In contrast, we defined 2 clusters of patients with high neutrophil levels: OAC2 (*n* = 12) patients have the highest levels of sputum neutrophils (91.3%, Table 1) and are predominantly female (75%) with high levels of allergic rhinitis and eczema while OAC4 (*n* = 12) patients are all severe asthmatics with the lowest incidence of allergic rhinitis (9%), lowest history of pneumonia (10%) and the second highest levels of sputum neutrophils (65.4%, Table 1). All patients in this group are either overweight or obese (Table 1). Finally, OAC5 consists of 10 patients with the highest incidence of allergic rhinitis (88%) and combined atopy based on the regional aeroallergens (80%).

### 3.3 Analysis of OAC clusters according to sputum granulocytes

Sputum granulocyte status is a recognised way to sub‐phenotype patients with asthma.^14^, ^20^ We found that there was a preferential enrichment of eosinophilic asthma in OAC3 (83%) and in patients in OAC4 (58%) with the least number of eosinophilic patients in OAC2 (15%) (Table 2). In contrast, OAC2 patients were preferentially enriched in the patients with neutrophilic and mixed granulocytic asthma. Of all OAC2 subjects, 41.7% (5 of 12) were neutrophilic and 50% (6 of 12) were mixed granulocytic phenotype. The OAC2 mixed group were represented by 3 subjects with nasal polyps and 3 patients with allergic rhinitis. We acknowledge that the higher prevalence of rhinitis in OA2 compared to OA3, while seemingly contradictory to previous studies, is clarified by the presence of mixed granulocytic subjects in OA2. We divided OA2 subjects into neutrophilic and mixed types. Overall, there were no significant clinical or biochemical/biomarker differences between the mixed granulocytic and neutrophilic subtypes in OAC2, which may reflect the low numbers (6 and 5) in each group (see Table S4). Patients with paucigranulocytic asthma represented the majority of patients in OAC1 and OAC5 clusters (Table 2).

**TABLE 2:** Distribution of sputum eosinophilic inflammation across OAC clusters.

						Group comparison (*p* values)									
	OAC1	OAC 2	OAC3	OAC4	OAC5	1 vs. 2	1 vs. 3	1 vs. 4	1 vs. 5	2 vs. 3	2 vs. 4	2 vs. 5	3 vs. 4	3 vs. 5	4 vs. 5
**Sputum eosinophilia, *n* (%)**	5 (25%)	1 (8.3%)	15 (83.3%)	7 (58.3%)	2 (20%)	<.001	**	***	***	ns	***	***	*	***	***
**Sputum neutrophilia, *n* (%)**	1 (5%)	5 (41.7%)	1 (5.6%)	2 (16.7%)	1 (10%)	.046	**	ns	*	ns	**	**	**	*	ns
**Sputum mixed, *n* (%)**	0 (0%)	6 (50%)	1 (5.6%)	1 (8.3%)	1 (10%)	.001	**	*	**	**	**	**	**	ns	ns
**Sputum pauci granulocytic, *n* (%)**	14 (70%)	0 (0%)	1 (5.6%)	2 (16.7%)	6 (60%)	<.001	***	***	***	ns	*	***	***	*	***

*Note*: Statistical significance: *p < .05; **p < .01; ***p < .001; ****p < .0001; otherwise nonsignificant (*p* > .05).

**FIGURE 2:**
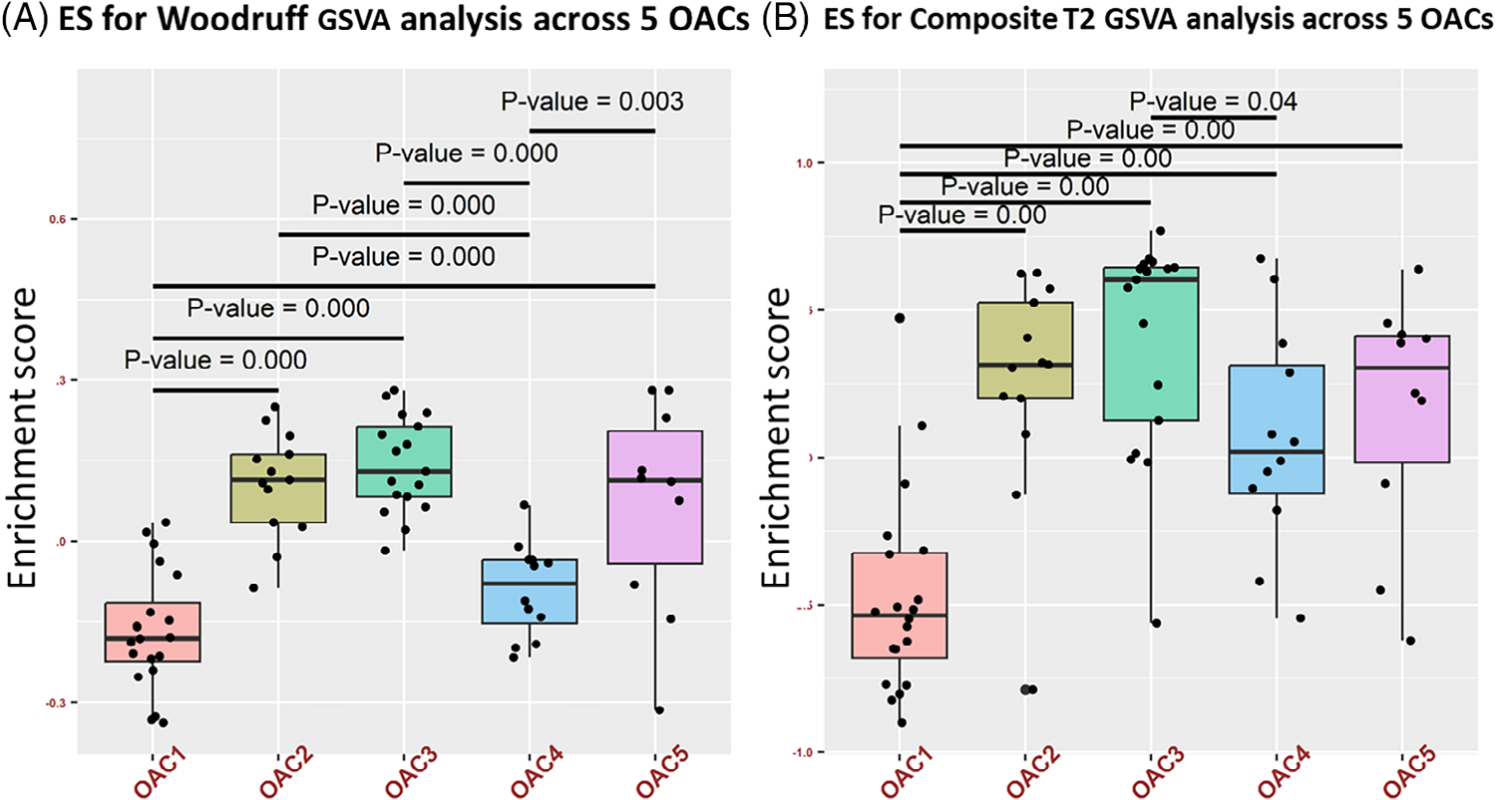
Enrichment of type 2 (T2) inflammation in omics‐associated cluster (OAC)3. Gene set variation analysis (GSVA) boxplots showing the enrichment score (ES) for the Woodruff T2 signature (A) and a composite T2 cytokine and mediator panel (B) in sputum omics‐associated cluster (OAC)3 compared to other OACs.

Molecular phenotyping of the OACs indicated that patients within the OAC3 cluster were enriched for the Woodruff T2 signature^21^ (Figure 2A) and for a composite of various T2‐specific mediators (Figure 2B) including IL‐5 (*p* = .00183, Figure S3A), IL‐13 (*p* = 5 × 10^−5^, Figure S3B) and IL‐4 (*p* = .05493, Figure S3C). Patients in OAC3 represent those with severe eosinophilic T2 asthma and have a similar comparative enrichment of the Woodruff signature as reported previously between the eosinophilic TAC1 and the neutrophilic/inflammasome‐containing TAC2 subjects.^14^ Data from the U‐BIOPRED cohort show that sputum eosinophil changes partially reflect changes in blood eosinophils, with the correlation strength affected by OCS use. OCS‐dependent subjects had a positive correlation with sputum–blood eosinophil (rho = .44), which was increased for OCS nondependent subjects (rho = .55). The correlation between OCS use and sputum neutrophils was significant only in OCS‐dependent subjects (rho = .31) and not in non‐OCS users.

### 3.4 Molecular analysis of OAC clusters according to asthma severity

To explore the molecular variances between MMA and SA in clusters, we conducted a comparative analysis of sputum omics datasets for OAC1. Our findings indicated that the MMA sample sizes in OAC2 (*n* = 1), OAC3 (*n* = 0), OAC4 (*n* = 0), and OAC5 (*n* = 3) were insufficient for meaningful analysis in these clusters. Based on initial univariate analysis comparing MMA with SA, we observed that confounding effect of clinical variables such as BMI, smoking status and atopy can influence gene expression levels and asthma severity. By adjusting for these variables, we can better isolate the true molecular differences between MMA and SA in OAC1 and reduce the influence of confounding factors. After adjusting for these confounding factors, we found a number of proteins being upregulated in the severe asthmatics compared to MMA. These were Complement C9, MIA, TIMP2, UNC5C, CFP, CCL21, KNG1, EFNA5, PRSS22, ADAM9, RETN, TNFRSF21, RARRES2, TXNDC12, EPHB2 and VEGFA (Figure S4).

We ran network analysis using these proteins and string‐db (version 12.0). By utilising these proteins and conducting a pathway analysis, we discovered the enrichment of several biological terms. Three gene ontology (GO) terms were overrepresented. The common elements between these three processes are the genes CCL21, TNFRSF21 and KNG1. These proteins are involved in the humoral immune response, negative regulation of cell adhesion, and response to external stimulus. Eleven GO compartments were overrepresented. Common genes among these compartments include RETN, CFP, TIMP2, RARRES2 and KNG1. These genes are involved in the extracellular region, extracellular space, secretory granule lumen and specific granule lumen, highlighting their roles in extracellular and granule‐related processes. Table S5 presents enriched biological terms and protein features associated with SA compared to MMA in OAC1.

### 3.5 Reduced microbial diversity in OAC2

**FIGURE 3:**
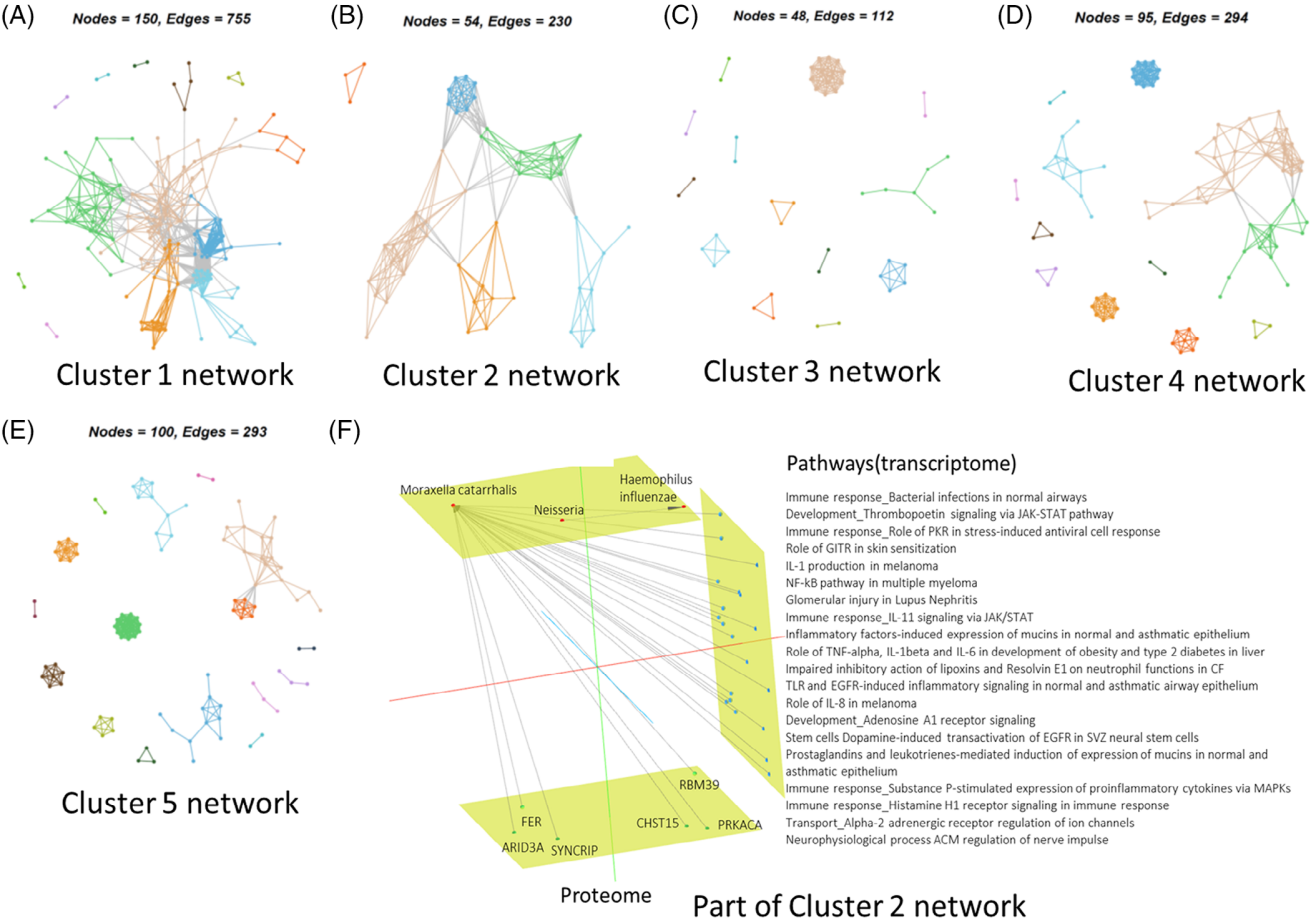
Network visualisation of clusters formed by interaction between omics and a multi‐channel view of omics interactions related to omics‐associated cluster (OAC)2. Communities in the multi‐omics data detected via random walks clustering for OAC1 (A), OAC2 (B), OAC3 (C), OAC4 (D) and OAC5 (E). (F) Highlights proteins and pathways that serve as distinctive signatures of OAC2, showcasing their interaction with microbiome signatures (*r* > .4) and are shown using the Arena3Dweb tool.

We next examined the microbial diversity across the 5 OACs (Figure S4). This analysis indicated that the Shannon index of alpha diversity from the metagenomics or 16S omics was significantly reduced in OAC2 (the high sputum neutrophil cluster) compared to the other clusters including OAC4 (Figure S4A and B). Network analysis identified distinct multi‐omic networks of nodes and edges in OAC1 (Figure 3A), OAC2 (Figure 3B), OAC3 (Figure 3C), OAC4 (Figure 3D) and OAC5 (Figure 3E). Further analysis of OAC2 demonstrates the association of upregulated gene and protein pathways in this cluster with a high enrichment of *Moraxella catarrhalis* and an association with *Haemophillus influenzae* (Figure 3F). In contrast, OAC1 was associated with the presence of commensal bacteria such as *Prevotella* and there was a limited association of upregulated pathways with any bacterial species for OAC3–5.

### 3.6 Pathways associated with OAC clusters

**FIGURE 4:**
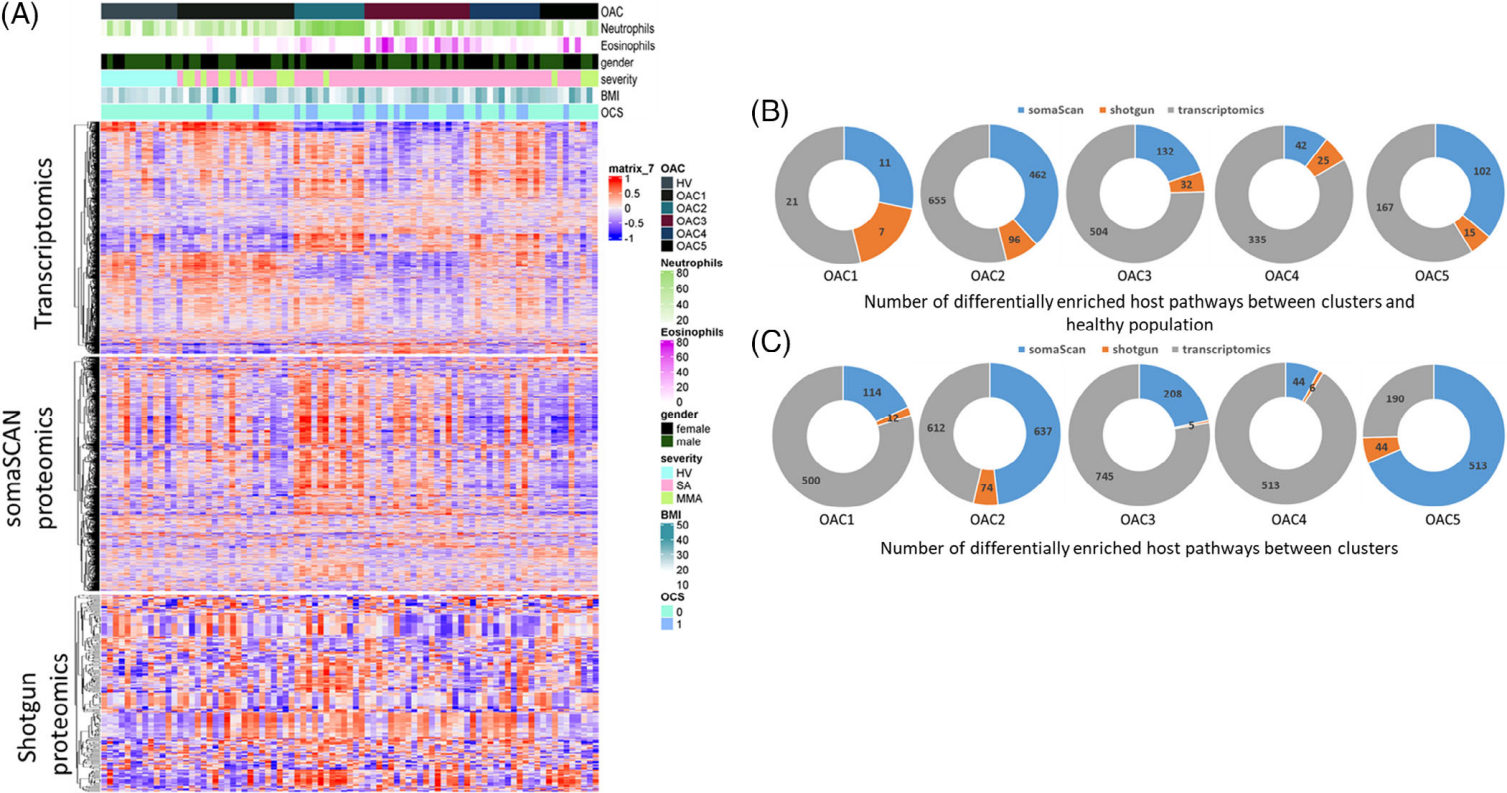
Enrichment of genes, proteins and pathways across the sputum omics‐associated clusters. (A) Heatmap of 72 asthmatic subjects (columns) with 1472, 1362 and 781 pathways (rows) based on the gene set variation analysis (GSVA) of transcriptomic, somaScan and shotgun proteomics datasets, respectively. The sputum neutrophil and eosinophil percentages, sex, oral corticosteroids (OCS) use, body mass index (BMI), and asthma severity for each participant are mapped above the Heatmap. (B) Piechart of number of differentially enriched pathways between clusters and healthy population (*q* value < .05). The numbers on the segments are number of differentially enriched pathways between the 5 omics‐associated clusters (OACs) and healthy volunteers (HV). (C) Pie chart of the number of differentially enriched pathways between clusters (*q* value < .05). The numbers on the segments show differentially enriched pathways by one versus comparison (e.g. OAC1 vs. OAC2:5). Pie charts are coloured based on the datasets: SomaScan in blue, shotgun proteomics in orange and transcriptomics in grey. SA, severe asthma; MMA, mild and moderate asthma.

Figure 4A shows the heatmap of the pathway enrichment scores for the transcriptomics, SomaScan proteomics and shotgun proteomics according to 5 OAC clusters compared with HV and the assignment of subjects according to sputum granulocyte status, gender, asthma severity, body mass index (BMI) and oral corticosteroid (OCS) therapy. We initially examined the number of significantly (*q* value < .05) differentially enriched pathways between HV and patients in each OAC (Figure 4B). There were 39, 1213, 668, 402, 284 pathways differentially enriched between HV and OAC1‐5, respectively. The top 10 pathways with most replication in the list of significantly differentiated pathways in the group comparison between healthy and OACs are shown in Table 3. No pathway was consistently perturbed between HV and the OACs. The most frequently enriched pathways across OAC1‐5 compared with HVs include those related to complement and platelet activation, cell adhesion and eosinophil and Th2 pathways (Table 3). Because we are using more than one *omics* dataset and several groups of subjects, one pathway can be detected more than once either by *omics* datasets or by groups. The differentially enriched pathways (DEPs) across all 3 *omics* datasets compared to HVs were limited to coagulation in OAC3 versus HVs and cell adhesion, platelets, immune responses and MIF in OAC2 compared with HVs (Table S6).

**TABLE 3:** Frequency of enriched pathways.

Pathways	Frequency
Blood coagulation Platelet microparticle generation	8
Cell adhesion Plasmin signalling	7
Cytoskeleton remodelling Keratin filaments	6
Development Transcriptional regulation of megakaryopoiesis	6
Eosinophil granule protein release in asthma	6
Immune response CCR3 signalling in eosinophils	6
Immune response LPS‐induced platelet activation	6
Platelet activation during ADAM‐TS13‐deficient thrombotic microangiopathy development	6
SHH signalling in colorectal cancer	6
Th2 cytokine‐ and TNF‐alpha‐induced inflammatory response in asthmatic airway fibroblasts	6

*Note*: The first column shows pathways, and the second column shows the frequency that these pathways were differentially enriched between healthy and clusters 1:5 based on the transcriptomics and proteomics datasets.

The 5 pathways with the highest and lowest median fold‐change for each comparison are shown in Table S7. All significantly enriched pathways are reported in the Supplementary file ‘SignificantlyDifferentWithHG.xlsx’. Transcriptomics data predominantly drove the total number of DEPs, particularly for OAC3 and 4 (Figure 4B). However, the top DEPs (up‐ and downregulated) between OACs and HV were driven by proteomic data for OAC1 (6/10 pathways), OAC2 (10/10 pathways) and OAC3 (7/10 pathways) (Table S7).

We then compared the significant (*q* value < .05) DEPs between each OAC (Figure 4C). Each pie chart shows the number of perturbed pathways between one OAC and the remaining OACs. We found 626, 1323, 958, 563 and 747 DEPs across OAC1–5, respectively, which is considerably higher than the number of perturbed pathways seen in comparison with HV. All pathways are reported in the Supplementary file ‘SignificantlyDifferentIntraOACs.xlsx’. Transcriptomic data drove most pathways for OAC1, 3 and 4 while proteomics drove the majority of pathways in OAC2 and 5 (Figure 4C).

For each cluster, the DEPs based on all three datasets are shown in Table 4. The top 10 pathways with most replication between groups are shown in Table 5. Table S8 shows the 5 pathways with highest and lowest median fold‐change. Thirty‐one pathways were commonly occurred across all the group comparison; this is shown in Table S9. Interestingly, we saw an initial reduction in the enrichment of the pathway ‘Eosinophil survival in Asthma’ in OAC3, which was surprising as we expected this to be enriched (Table S8). Further analysis of the up‐ and downregulated genes in this pathway indicated an upregulation of the positive genes in OAC3 and downregulation of the negative genes in this pathway (Figure S6). This indicates the need to further analyse pathways that include both up‐ and downregulated genes.

**TABLE 4:** Differentially enriched pathways (up‐ or downregulated) from all three omics datasets between each omics‐associated cluster (OAC) versus the rest.

Omics‐associated clusters (OAC)	Pathways identified by all three omics datasets
**OAC1**	Cell adhesion; Plasmin signalling
	Eosinophil adhesion and transendothelial migration in asthma
**OAC2**	Development Regulation of endothelial progenitor cell differentiation from adult stem cells
	Development Regulation of epithelial‐to‐mesenchymal transition (EMT)
	Eosinophil adhesion and transendothelial migration in asthma
	Inhibition of neutrophil migration by proresolving lipid mediators in COPD
	Nociception Nociceptin receptor signalling
	Prolactin/ JAK2 signalling in breast cancer
	Proteases and EGFR‐induced mucin synthesis in normal and asthmatic epithelium
	Role of cell adhesion in vaso‐occlusion in Sickle cell disease
	Role of integrins in eosinophil degranulation in asthma
	Role of platelets in allograft rejection
	Role of platelets in the initiation of in‐stent restenosis
**OAC3**	Role of IL‐23/ T17 pathogenic axis in psoriasis
**OAC4**	None
**OAC5**	Development Keratinocyte differentiation
	ErbB2‐induced breast cancer cell invasion
	Immune response IL‐3 signalling via ERK and PI3K

**TABLE 5:** Differentially enriched pathways of the transcriptomics and proteomics datasets that occur frequently between OACs.

Differentially enriched pathways	Frequency
Role of integrins in eosinophil degranulation in asthma	8
Blood coagulation Blood coagulation	7
Cell adhesion Plasmin signalling	7
Development Keratinocyte differentiation	7
Development Leptin signalling via JAK/STAT and MAPK cascades	7
Development VEGF‐family signalling	7
Eosinophil adhesion and transendothelial migration in asthma	7
ErbB2‐induced breast cancer cell invasion	7
Immune response IL‐3 signalling via ERK and PI3K	7
Mechanisms of drug resistance in SCLC	7

Figure S7 presents a word cloud visualization of genes involved in the 31 common pathways. Each word represents a gene symbol, with its font size increasing based on the number of pathways it participates in, thereby indicating its relative importance. It highlights the key proinflammatory pathways such as MAPK, PI3K‐AKT, NF‐κB and apoptosis across the OACs with the 2 most important genes being AKT2 and MAPK1 which occur in 18 pathways. OAC1 was associated with apoptosis and immune responses, OAC2 with cell adhesion and neutrophil function, OAC3 with Wnt signalling and MAPK activation in asthma, OAC4 with checkpoint and NF‐κB activation and OAC5 with glucocorticoid and growth factor activation.

We next analysed the 5 OACs according to ActivePathways.^16^ This methodology integrates multiple molecular datasets and pathway annotations to enhance systems‐level understanding of cellular organisation in health and disease. OAC1 was linked to NEF‐mediated transport linked to T‐cell receptor signalling and antigen‐presenting cells (Figure S8) and OAC2 with bile metabolism and fatty bile metabolism (Figure S9). This suggested that patients in OAC2 may have suffer from gastro‐oesophageal reflux disease (GORD) because of the link between abnormal bile metabolism and GORD.^22^ Indeed, 7 out of the 12 OAC2 subjects have either active GORD or have previously used GORD medication. Patients with OAC3 were predominantly associated with AMPK activation reflecting a metabolic imbalance (Figure S10) while OAC5 was associated with extracellular matrix degradation, cell junction formation and plasma assembly remodelling (Figure S11). There were no ActivePathways identified for OAC4.

### 3.7 IL‐22 activation in OACs – a new method for drug repurposing

We then utilised a computational method to find drugs that may be associated with patients in OAC4. This approach was to re‐purpose existing drugs for asthma using gene signatures that reflect transcriptomic as well as clinical response to therapies. Thus, we examined whether there was a pattern of (1) downregulation of disease downregulated genes, (2) upregulation of disease upregulated genes, (3) downregulation of genes upregulated by the drug, and (4) upregulation of genes downregulated by the drug. Enrichment scores of the disease and drug signatures of those 4 aspects were plotted for each cluster. By joining the average enrichment scores for each signature, the cluster that follows the pattern shows an N shape, hence the name ‘N’ method for this stratification. We examined the drug, fezakinumab (FZ), which is an anti‐IL22 antibody that blocks the effect of the IL‐22 cytokine that has been approved for the treatment of atopic dermatitis (AD).^23^

**FIGURE 5:**
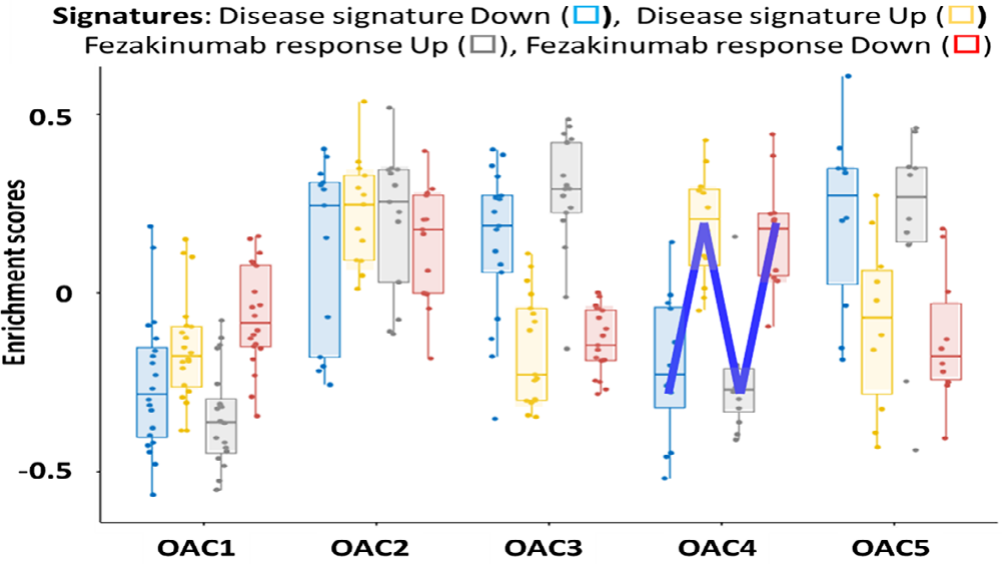
Gene set variation analysis (GSVA) of disease and anti‐IL‐22 (Fezakinumab, Fz) response signatures across 5 omics‐associated clusters (OACs)1‐5. Significantly down‐ and upregulated genes expressed in disease (Disease signature Down □ and Up □), and genes that are significantly up‐ and downregulated by Fz (Fz response Up □, FZ response Down □) are shown for OACs 1–5. A cluster that is likely to respond to Fz will have a low Disease Down and Fz Up signature enrichment and a high Disease Up and Fz Down signature. This results in an ‘N’‐shaped response (indicated in blue). This criterion is met by OAC4.

We investigated four signatures derived from AD patients: two AD disease signatures (one up and one down) and two signatures of lesional AD skin tissue following FZ therapy (one up and one down).^24^ OAC4 patients gave an N‐shaped response suggesting that these subjects are most likely to respond to FZ (Figure 5). In contrast, using the same approach only 2, 3, 0 and 1 subject(s) from OAC1, 2, 3 and 5 respectively could be categorised as potential responders.

### 3.8 Comparison between neutrophilic subtypes in OAC2 and OAC4

**FIGURE 6:**
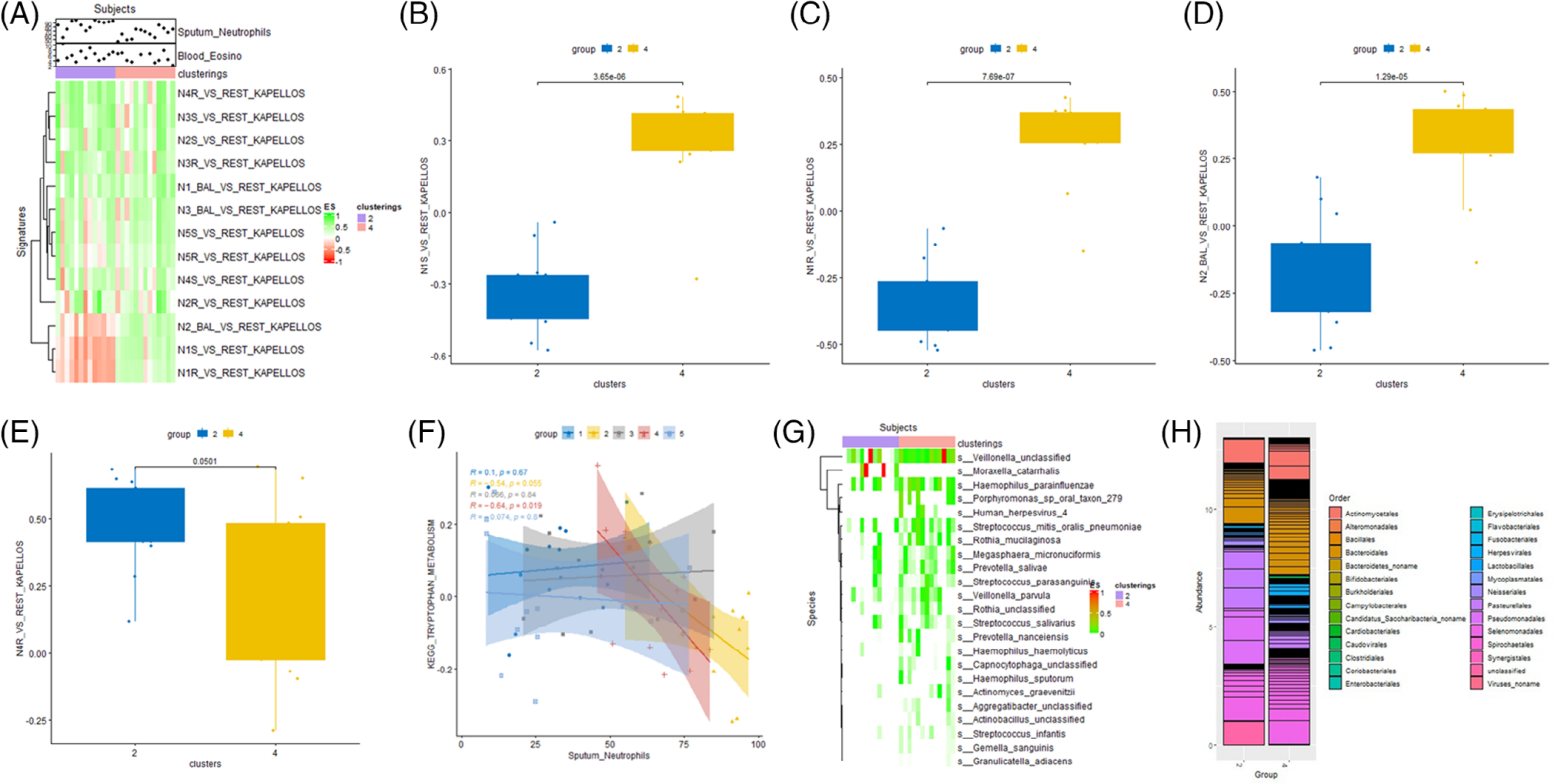
Differential enrichment of neutrophil subsets between neutrophil‐enriched omic‐associated clusters (OAC2) and OAC4. (A) Heatmap showing enrichment of neutrophil subtype gene signatures according to OAC2 and 4 and correlation with blood eosinophils and sputum neutrophil percentages. Boxplots showing enrichment scores (ES) for gene set variation analysis (GSVA) of neutrophil subtypes N1S (B), N1R (C), N2BAL (D) and (N4R (E) in the sputum of OAC2 and OAC4 patients. Adjusted *p* values are shown. (F) Inverse relationship between sputum neutrophil percentages and tryptophan metabolism in OAC4 (*r* = −.64, *p* = .019) but not in other OACs. (G, H) Heatmaps of microbial species showing the increased prevalence of Moraxella catarrhalis in OAC2 compared to OAC4 and a greater reduction of most other species in OAC4 compared to OAC2.

Since we obtained two distinct neutrophilic subtypes in our cluster analysis, we utilised GSVA to undertake cellular deconvolution to identify whether different neutrophil subtypes^25^ were present in the two OAC clusters. Clustering of the enrichment scores for the 3 BAL neutrophil subtypes and the 5 peripheral blood neutrophil subsets identified by either Seq‐Well (S) or Rhapsody (R) (Figure 6A) demonstrated significant enrichment of neutrophil subtypes between OACs (Figures 6A and S12). There was a significant enrichment of the LCN2 expressing neutrophil progenitors N1S (Figure 6B) and N1R (Figure 6C) cells and of the N2BAL (Figure 6D) neutrophil subset, enriched for genes related to influenza infection, in OAC4 compared to OAC2. No neutrophil subsets were significantly enriched in OAC2 compared with OAC4 although that of the endpoint N4R neutrophil subset almost reached significance (*p* = .051, Figure 6E). There was no significant difference in the expression of other intermediate or endpoint neutrophil subsets or in the N1BAL and N3BAL subsets. In addition, there was an inverse relationship between sputum neutrophil percentages and tryptophan metabolism in OAC4 (*r* = −.64, *p* = .019) but not in other OACs (Figure 6F). As we have previously shown a reduction in Shannon Index in OAC2 compared with OAC4 (Figure S5), we examined the relative expression of microbial species between these 2 clusters. A heatmap of microbial species highlights the prevalence of *Moraxella catarrhalis* in OAC2 compared to OAC4 and a greater reduction of most other species in OAC4 compared to OAC2 (Figure 6G and H).

### 3.9 Neutrophilic activation mechanism between neutrophilic subtypes in OAC2 and OAC4

To further investigate the neutrophilic activation mechanisms in OAC2 and OAC4, we analysed 129 signatures related to various aspects of neutrophil activation and aging, as well as Th1‐, Th17‐, ILC3‐, macrophage‐, NETosis‐ and MyD88‐related signatures. We examined the enrichment scores of these signatures in OAC2 and OAC4 using GSVA, applying a significance threshold of α = .05. The 129 pathways are detailed in the Table S10, along with their statistical significance between the OAC2 and OAC4 groups and comparisons to healthy controls.

OAC2 shows heightened neutrophil functions linked to IgE‐dependent proinflammatory mediator release, chemotaxis, activation, apoptosis, NETosis and immune response versus HV and OAC4. Contrastingly, OAC4 cluster displays enrichment for neutrophil activation and aging including Th17‐, TLR‐ and MyD88‐related signatures compared to OAC2. In addition, macrophage signatures suggested that M1 drivers such as IFN‐γ, TNF, LPS and fatty acid stimulation were significantly enriched in OAC4 compared with OAC2.

## 4 DISCUSSION

Integration of sputum multi‐omics data identified 5 clusters with distinct clinical and pathway features which mapped to some asthma clinical subtypes including a severe asthma cluster with high sputum eosinophilia and OCS use (OAC3). OAC3 patients were enriched for the expression of T2 cytokines, mediators and signatures. Patients with paucigranulocytic asthma represented the majority of patients in OAC1 and OAC5 clusters. OAC1 consists mostly of MMA at 55%, compared to OAC5's 27% MMA composition. Prolonged asthma duration, smoking, allergic rhinitis, and allergy status likely shape the molecular profiles of OAC1 and OAC5 patients, aligning them more closely with severe asthma (SA) cases. Elevated proteins and pathways in SA compared MMA in OAC1 reveal processes involving stronger humoral immune responses, altered cell adhesion, increased reactivity to external stimuli, and significant extracellular and secretory activity. These differences highlight the complex interplay between clinical phenotypes (MMA vs. SA) and underlying molecular mechanisms in asthma pathogenesis.

Importantly, we identified 2 sputum neutrophil clusters – those with the highest levels of sputum neutrophilia (OAC2) were associated predominantly with the presence of *Moraxella catarrhalis* and to a lesser extent with *Haemophilus influenzae* and a low α‐diversity and were also present in the mixed granulocytic group. By contrast, the other sputum neutrophil high group (OAC4) were all severe asthmatics and were associated with IL‐22 activation rather than the presence of pathogenic bacteria. A subset of these subjects was also present in the sputum eosinophil high group. Furthermore, we show that OAC2 and OAC4 were associated with distinct subtypes of neutrophils and inversely associated with tryptophan metabolism. Thus, the application of a multi‐omics analysis to severe asthma identified molecular phenotypes not distinguished by clinical and biochemical features, but involving the presence of distinct neutrophil subtypes that may respond to different therapeutic approaches.

Across various aspects of neutrophil activation and aging, as well as Th1‐, Th17‐, ILC3‐, macrophage‐, and MyD88‐related signatures, OAC4 exhibits a higher level of enrichment for activated aging neutrophils influenced than OAC2. This increase is associated with the activation of TLR and MyD88 pathways. Overall, Th17 signatures were more enriched in OAC4 than in OAC2 patients although no clear differences were seen for Th1 and ILC3 signatures. In addition, macrophage signatures suggested that M1 drivers such as IFN‐γ, TNF, LPS and fatty acid stimulation were significantly enriched in OAC4 compared with OAC2. In contrast, the neutrophil signature associated with IgE‐dependent proinflammatory mediator release is more enriched in OAC2 than in OAC4 and may reflect the increased degree of atopy and atopy‐related diseases seen in patients in the OAC2 cluster. A NETosis signature was also more enriched in OAC2 than in OAC4.

Although most intermediate and endpoint neutrophil subtypes^25^ were similar between the OAC2 and OAC4 groups, there were some differences identified. The LCN2‐containing N1 progenitor neutrophil signature, which was enriched in the OAC4 group, also contains the alarmins (S100A8, S100A9, S100A12) that drive mucus hypersecretion, alveolar destruction and lung function decline in COPD.^26^, ^27^ In addition, N2BAL neutrophils, which were significantly enriched in OAC4 compared to OAC2 patients, preferentially express genes associated with influenza virus infection and translation.^25^ Overall, these differences in neutrophil subtypes present between the two neutrophil high groups may reflect the different levels of pathogenic microbial species present and/or ongoing viral infection in OAC4 patients. Any infection in subjects, whether bacterial or viral, would have been subclinical as sputum induction was not performed within 3 months of an exacerbation.^28^

A link between sputum neutrophilia and airway bacterial imbalance exists which suggests that patients with this phenotype of asthma have an underlying molecular phenotype reflecting the host‐microbial innate immune response and changes in the expression of protective mechanisms.^29^ Analysis of the sputum metagenome in the U‐BIOPRED cohort indicated a lower α‐diversity at the species level in patients with severe asthma compared to mild‐to‐moderate asthma and healthy controls.^15^ The α‐diversity was also decreased in the previously described transcriptome‐associated clusters (TACs),^14^ with TAC1 and TAC2 subjects having high levels of *Haemophilus influenzae* and *Tropheryma whipplei* and *Haemophilus influenzae* and *Moraxella catarrhalis*, respectively, compared to healthy controls. Sputum neutrophil counts correlated with *Moraxella catarrhalis*.^15^ In addition, *Haemophilus influenzae* has been commonly cultured and sequenced in a separate small cohorts of stable patients with severe sputum neutrophilic asthma.^30^

Two clusters of adult patients with severe asthma were defined by the sputum microbiome within the U‐BIOPRED study.^11^ Patients within the lowest Bray–Curtis microbiome β‐diversity had relatively worse asthma outcomes, raised sputum neutrophils and a microbial imbalance with elevated levels of the pathogenic *Haemophilus influenzae* and *Moraxella catarrhalis*.^11^ Subsequent analysis of the multi‐omics profiles associated with these two severe asthma clusters identified many differentially expressed genes, proteins and eicosanoids.^31^ These emphasised the importance of innate immune dysregulation, inflammation, toll receptor activation, IFN‐/Th1‐/Th17‐related pathways and neutrophil activation in the more severe pathogenic bacteria‐containing cluster.^31^

IL‐22 is released by several immune cells such as CD4+ T helper cells, γδT cells, NK cells and ILC3 cells and is implicated in the pathophysiology of various chronic inflammatory diseases including asthma and COPD and in mucosal‐associated infections.^24^, ^32^ The IL‐22 receptor (IL‐22R) is localised to structural cells, particularly epithelial cells, within the airway allowing immune‐stromal cell crosstalk and the regulation of antimicrobial proteins and neutrophil chemoattractants. IL‐22 also promotes epithelial proliferation and repair following injury.^33^ IL‐22 levels are increased in children and adults with allergic airway diseases and is likely to be associated with proinflammatory features.^34^ For example, a cross‐sectional study of asthmatic patients with highly neutrophilic asthma identified high levels of IL‐17+ and IL‐22+ staining cells in the bronchial lamina propria compared to patients without neutrophilic asthma.^35^ In addition, IL‐22 might control the extent of IFN‐γ‐mediated lung inflammation and therefore play a tissue‐restricted regulatory role.^36^ We have previously reported that an IL‐22/Th22 signalling pathway gene signature was raised in patients with severe neutrophilic and mixed granulocytic asthma. These patients were also enriched for a 296 gene signature obtained from atopic dermatitis patients who were clinical super‐responders to the anti‐IL‐22 antibody, Fezakinumab.^24^

In a mouse model of neutrophilic asthma generated by OVA‐LPS challenge, neutrophilia was associated with raised levels of IL‐17A and IL‐22 all of which were attenuated following ILC depletion using Rag2^–/–^ mice.^37^ In a similar model, repeated low dose nasal endotoxin challenge enhanced AHR, BAL neutrophilia and elevated levels of IL‐17A and IL‐22.^38^ Furthermore, the combination of IL‐22 with IL‐17, but neither alone, elicited AHR in naïve mice and both elicited proinflammatory mediator release from primary HBECs. RORγt blockade in a Th2 low mouse model of asthma suppressed both IL‐17 and IL‐22 expression and attenuated AHR and neutrophilia.^39^ In contrast, some mouse studies suggest that IL‐22 may have both inflammatory and anti‐inflammatory effects in asthma^40^ since administration of exogenous IL‐22 plays a protective role in an OVA‐induced asthma model.^41^ A similar bidirectional effect of IL‐22 is seen in mouse models of COPD^42^ although we have previously reported elevated levels of IL‐22 and IL‐22R mRNA and protein in the airways of mild‐to‐moderate COPD patients. In a mouse model pulmonary neutrophilic inflammation, airway remodelling and emphysema were reduced and lung function was improved in IL‐22 KO mice exposed to cigarette smoke compared to WT controls.^32^

In a similar sized study to the one reported here for severe asthma, Yan and coworkers^43^ examined multi‐omics integration of the sputum metagenome, metabolome, host transcriptome and proteome in COPD (99 subjects) compared with healthy control subjects (*n* = 36). There was a much greater host‐microbial association between neutrophilic compared to eosinophilic COPD.^43^ This confirms the results seen between the microbiome and the host transcriptome/proteome in this study whereby the neutrophilic OAC2 cluster has much more intense links to the microbiome than the eosinophilic OAC3 cluster. Interestingly, 3 metagenomic/host metabolomic features were associated with neutrophilic COPD with low levels of indole‐3‐acetic acid linked to reduced levels of IL‐22 pathway signalling in response to reduced microbial metabolism.^43^ The presence of different levels and types of bacteria between OAC2 and OAC4 may explain the difference in IL‐22 association with neutrophilia seen in this study. The presence of *Moraxella catarrhalis* and, to a lesser extent*, Haemophilus influenzae* in OAC2 may have clinical implications. Azithromycin attenuated airway *Haemophilus influenza* burden in persistent uncontrolled asthma without affecting total or *Moraxella catarrhalis* bacterial load in the Asthma and Macrolides: The Azithromycin Efficacy and Safety (AMAZES) trial.^44^ Greater activation of Th17 pathways in OAC4 compared to OAC2 (Table S10) might explain the lack of microbial dysbiosis observed in OAC4 since the Th17 pathway has antimicrobial effects. In addition, IL‐22 is also produced by Th‐17 cells. Clinically, this suggests that patients in OAC4 might have a preferential response to anti‐IL‐22 therapy while those associated with OAC2 might be better treated with antibiotics such as macrolides that may suppress Haemophilus influenzae and Moraxella catarrhalis.

Multi‐omics analysis is increasingly recognised as a crucial tool in asthma research due to its comprehensive approach to investigating the disease's multifaceted nature.^45^, ^46^ By incorporating diverse omic fields such as genomics, transcriptomics and proteomics, it unveils the complex molecular interactions at play in asthma's pathogenesis and progression. Crucially, multi‐omics facilitates the discovery of new biomarkers and the refinement of disease phenotyping and endotyping, paving the way for personalised therapies.^45^, ^46^ Despite its potential, the full integration of omics‐derived insights into clinical settings remains a work in progress, underscoring the necessity for continued research to bridge the gap between scientific discovery and therapeutic application.

Li et al.^47^ used SNF and integrated nine omics data blocks from various anatomical sites in 52 women, enhancing group classification accuracy for COPD patients versus healthy never‐smokers and smokers with normal spirometry. This reduced the necessary group sizes from 30 to 6 for 95% power, with four to seven omics combinations achieving over 95% accuracy. By enhancing classification accuracy and reducing the need for larger sample sizes, this method addresses the issue of detecting significant findings with smaller groups, mitigating the risk of type I and type II errors commonly associated with inadequate sample sizes.

Niazi^48^ highlighted FDA's increasing emphasis on omics technologies; the future of the field lies in embracing a multi‐omics perspective. While Niazi et al.^48^ underscore the potential of individual omics approaches, integrating these diverse datasets holds the key to unlocking a more comprehensive understanding of complex biological systems.

Although we acknowledge that larger, more comprehensive studies are needed to fully realise the potential of multi‐omics integration, the study conducted here thus far serves as crucial pilot and scoping exercises. This initial foray into integrated omics provides a valuable foundation for future research and paves the way for larger‐scale investigations. By embracing this powerful approach, we can drive the field, leading to more efficient drug development and improved patient access to life‐changing therapies.

There are several limitations to this study including the lack of a replication cohort. As far as we are aware there are no publicly available asthma or severe asthma datasets that incorporate this depth of *omics* analysis. Asthma is a dynamic condition with variable disease trajectories.^49^ Clusters, if over only one time point, may not capture the evolving nature of the disease over time and patients may transition between different phenotypes. Our identified clusters were mostly based on the UK and European Caucasian population and might not generalise well across diverse populations. Factors such as geographic location, ethnicity and environmental exposures can influence the manifestation of asthma and may limit the generalisability of findings to broader populations. Small clusters may lead to overfitting and clusters may not generalise well to other populations. In addition, we did not undertake bacterial culture to confirm the bacterial species reported here. Although we were previously able to demonstrate enhanced expression of sputum IL‐22 protein and the IL‐22/Fezakinumab gene response signature in patients with severe asthma,^24^ the numbers in OAC clusters 2 and 4 did not generate significant differences in this study. Finally, we may have identified more OAC‐associated genes and proteins if we had used bulk or single cell RNA‐sequencing together with a higher detection of proteins.

Our workflow integrating sputum omics data has generated interesting clusters of asthma patients that make sense clinically and also provides novel insight into potential underlying pathways driving disease. The improved granularity obtained by fusing datasets probably results from not having to place subjects into an inappropriate bin as often happens if using a single omics dataset.^47^, ^50^ In a previous study combining sputum and serum datasets in asthma we found that addition of serum proteomics revealed heterogeneity in the neutrophil cluster; that is, it was divided into 2 subgroups.^50^ Including the metagenomics data, we have shown the presence of 2 neutrophil clusters in severe asthma sputum that are associated with distinct neutrophil subtypes, the presence or not of pathogenic bacterial species and identified a potential novel therapeutic target for a subset of patients with neutrophilic asthma.

## AUTHOR CONTRIBUTIONS

Ian M. Adcock and Kian Fan Chung conceived the idea; Ian M. Adcock and Kian Fan Chung obtained the funding for U‐BIOPRED project; Nazanin Zounemat Kermani, Ian M. Adcock and Kian Fan Chung discussed the approach to data analysis; Nazanin Zounemat Kermani and Ian M. Adcock analysed the data; Nazanin Zounemat Kermani, Ian M. Adcock and Kian Fan Chung wrote the manuscript; Nazanin Zounemat Kermani, Chuan‐Xing Li, Ali Versi, Yusef Badi, Kai Sun, Mahmoud I Abdel‐Aziz, Martina Bonatti, Anke‐Hilse Maitland‐van der Zee, Ratko Djukanovic, Åsa Wheelock, Sven‐Erik Dahlen, Peter Howarth, Yike Guo, Kian Fan Chung and Ian M. Adcock contributed to its finalisation and all authors agreed with the final version for submission. All authors gave final approval of the manuscript, had full access to all the data in the study, and had final responsibility for the decision to submit for publication.

## CONFLICT OF INTEREST STATEMENT

Mrs. Zounemat‐Kermani has nothing to declare. Dr. Maitland‐van der Zee has received grants from Health Holland and she is the PI of a P4O2 (Precision Medicine for more Oxygen) public–private partnership sponsored by Health Holland involving many private partners that contribute in cash and/or in kind (Boehringer Ingelheim, Breathomix, Fluidda, Ortec Logiqcare, Philips, Quantib‐U, Smartfish, SODAQ, Thirona, TopMD and Novartis), received unrestricted research grants from GSK, Boehringer Ingelheim and Vertex, received consulting fees paid to her institution from Boehringer Ingelheim and AstraZeneca, and received honoraria for lectures paid to her institution from GlaxoSmithKline – outside the submitted work. Dr. Dahlén reports personal fees from AZ, Cayman Chemicals, GSK, Novartis, Regeneron, Sanofi, TEVA, outside the submitted work. Dr. Chung has received honoraria for participating in Advisory Board meetings of Roche, Merck, Shionogi and Rickett‐Beckinson and has also been renumerated for speaking engagements for Novartis and AZ. Dr. Djukanovic declares consulting fees from Synairgen, Sanofi and Galapagos, lecture fees from GSK, AZ and Airways Vista and he holds shares from Synairgen. Dr. Li, Mr. Versi, Dr. Badi, Dr. Sun, Dr. Abdel‐Aziz and Mrs. Bonatti have nothing to declare.

## ETHICS STATEMENT

All recruited participants provided written informed consent, and local medical ethics committee approval was obtained by each study center. The study is registered at ClinicalTrials.gov under the identifier NCT01976767.

## Supporting information

Supporting Information

Supporting Information

Supporting Information

Supporting Information

Supporting Information

## Data Availability

The metagenomic sequence data have been submitted to the NCBI under accession number PRJNA946921 and are accessible at the following link: https://www.ncbi.nlm.nih.gov/sra/PRJNA946921. The sputum transcriptomics data have been submitted to the NCBI under accession number GSE76262 and are accessible at the following https://www.ncbi.nlm.nih.gov/geo/query/acc.cgi?acc = GSE76262.

## References

[1] Custovic A , Henderson J , Simpson A . Does understanding endotypes translate to better asthma management options for all? J Allergy Clin Immunol. 2019;144(1):25‐33. doi:10.1016/j.jaci.2019.05.016 Epub May 27 31145940

[2] Holguin F , Cardet JC , Chung KF , et al. Management of severe asthma: a European Respiratory Society/American Thoracic Society guideline. Eur Respir J. 2020;55(1):1900588. doi:10.1183/13993003.00588-2019 Print 2020 Jan 31558662

[3] Papi A , Brightling C , Pedersen SE , Reddel HK . Asthma. Lancet. 2018;391(10122):783‐800. doi:10.1016/S0140-6736(17)33311-1 Epub 2017 Dec 19 29273246

[4] Pavord I , Bahmer T , Braido F , et al. Severe T2‐high asthma in the biologics era: European experts' opinion. Eur Respir Rev. 2019;28(152):190054. doi:10.1183/16000617.0054-2019 Print 2019 Jun 30 31285291 PMC9489011

[5] Buhl R , Bel E , Bourdin A , et al. Effective management of severe asthma with biologic medications in adult patients: a literature review and international expert opinion. J Allergy Clin Immunol Pract. 2022;10(2):422‐432. doi:10.1016/j.jaip.2021.10.059 Epub Nov 8 34763123

[6] Chung KF . Type‐2‐low severe asthma endotypes for new treatments: the new asthma frontier. Curr Opin Allergy Clin Immunol. 2023;23(3):199‐204. doi:10.1097/ACI.0000000000000899 Epub 2023 Apr 12 37185823

[7] Hinks TSC , Levine SJ , Brusselle GG . Treatment options in type‐2 low asthma. Eur Respir J. 2021;57(1):2000528. doi:10.1183/13993003.00528-2020 Print 2021 Jan 32586877 PMC7116624

[8] Moore WC , Hastie AT , Li X , et al. Sputum neutrophil counts are associated with more severe asthma phenotypes using cluster analysis. J Allergy Clin Immunol. 2014;133(6):1557‐1563. 24332216 10.1016/j.jaci.2013.10.011 PMC4040309

[9] Kuo CS , Pavlidis S , Loza M , et al. A transcriptome‐driven Analysis of epithelial brushings and bronchial biopsies to define asthma phenotypes in U‐BIOPRED. Am J Respir Crit Care Med. 2017;195(4):443‐455. doi:10.1164/rccm.201512-2452OC 27580351

[10] Schofield JPR , Burg D , Nicholas B , et al. Stratification of asthma phenotypes by airway proteomic signatures. J Allergy Clin Immunol. 2019;144(1):70‐82. doi:10.1016/j.jaci.2019.03.013 Epub Mar 28 30928653

[11] Abdel‐Aziz MI , Brinkman P , Vijverberg SJH , et al. Sputum microbiome profiles identify severe asthma phenotypes of relative stability at 12 to 18 months. J Allergy Clin Immunol. 2021;147(1):123‐134. doi:10.1016/j.jaci.2020.04.018 Epub Apr 28 32353491

[12] Bunyavanich S , Schadt EE . Systems biology of asthma and allergic diseases: a multiscale approach. J Allergy Clin Immunol. 2015;135(1):31‐42. doi:10.1016/j.jaci.2014.10.015 Epub Nov 21 25468194 PMC4289105

[13] Shaw DE , Sousa AR , Fowler SJ , et al. Clinical and inflammatory characteristics of the European U‐BIOPRED adult severe asthma cohort. Eur Respir J. 2015;46(5):1308‐1321. 26357963 10.1183/13993003.00779-2015

[14] Kuo CS , Pavlidis S , Loza M , et al. T‐helper cell type 2 (Th2) and non‐Th2 molecular phenotypes of asthma using sputum transcriptomics in U‐BIOPRED. Eur Respir J. 2017;49(2). (pii):49/2/1602135. doi:10.1183/13993003.02135-2016 Print 2017 Feb 28179442

[15] Versi A , Ivan FX , Abdel‐Aziz MI , et al. Haemophilus influenzae and Moraxella catarrhalis in sputum of severe asthma with inflammasome and neutrophil activation. Allergy. 2023;7(10):15776. 10.1111/all.15776 37287344

[16] Paczkowska M , Barenboim J , Sintupisut N , et al. Integrative pathway enrichment analysis of multivariate omics data. Nat Commun. 2020;11(1):735. doi:10.1038/s41467-019-13983-9 32024846 PMC7002665

[17] Tibshirani R , Hastie T , Narasimhan B , Chu G . Diagnosis of multiple cancer types by shrunken centroids of gene expression. Proc Natl Acad Sci USA. 2002;99(10):6567‐6572. doi:10.1073/pnas.082099299 12011421 PMC124443

[18] Pons P , Latapy M , editors. Computing communities in large networks using random walks. Computer and Information Sciences – ISCIS 2005; 2005 2005//; Berlin, Heidelberg: Springer Berlin Heidelberg.

[19] Kokoli M , Karatzas E , Baltoumas FA , et al. Arena3D(web): interactive 3D visualization of multilayered networks supporting multiple directional information channels, clustering analysis and application integration. NAR Genom Bioinform. 2023;5(2):lqad053. doi:10.1093/nargab/lqad053 eCollection 2023 Jun 37260509 PMC10227371

[20] Simpson JL , Scott R , Boyle MJ , Gibson PG . Inflammatory subtypes in asthma: assessment and identification using induced sputum. Respirology. 2006;11(1):54‐61. 16423202 10.1111/j.1440-1843.2006.00784.x

[21] Woodruff PG , Modrek B , Choy DF , et al. T‐helper type 2‐driven inflammation defines major subphenotypes of asthma. Am J Respir Crit Care Med. 2009;180(5):388‐395. 19483109 10.1164/rccm.200903-0392OC PMC2742757

[22] Tariq K , Schofield JPR , Nicholas BL , et al. Sputum proteomic signature of gastro‐oesophageal reflux in patients with severe asthma. Respir Med. 2019;150:66‐73. (doi):10.1016/j.rmed.2019.02.008 Epub Feb 11 30961953

[23] Brunner PM , Pavel AB , Khattri S , et al. Baseline IL‐22 expression in patients with atopic dermatitis stratifies tissue responses to fezakinumab. J Allergy Clin Immunol. 2019;143(1):142‐154. doi:10.1016/j.jaci.2018.07.028 Epub Aug 17 30121291

[24] Badi YE , Pavel AB , Pavlidis S , et al. Mapping atopic dermatitis and anti‐IL‐22 response signatures to type 2‐low severe neutrophilic asthma. J Allergy Clin Immunol. 2022;149(1):89‐101. doi:10.1016/j.jaci.2021.04.010 Epub Apr 20 33891981

[25] Kapellos TS , Baßler K , Fujii W , et al. Systemic alterations in neutrophils and their precursors in early‐stage chronic obstructive pulmonary disease. Cell Rep. 2023;42(2211‐1247 (Electronic)):112525. 37243592 10.1016/j.celrep.2023.112525 PMC10320832

[26] Kang JH , Hwang SM , Chung IY . S100A8, S100A9 and S100A12 activate airway epithelial cells to produce MUC5AC via extracellular signal‐regulated kinase and nuclear factor‐κB pathways. Immunology. 2015;144(1):79‐90. doi:10.1111/imm.12352 24975020 PMC4264912

[27] Railwah C , Lora A , Zahid K , et al. Cigarette smoke induction of S100A9 contributes to chronic obstructive pulmonary disease. Am J Physiol Lung Cell Mol Physiol. 2020;319(6):L1021‐L1035. Epub 2020 Sep 23. doi:10.1152/ajplung.00207.2020 32964723 PMC7938777

[28] Shaw DE , Sousa AR , Fowler SJ , et al. Clinical and inflammatory characteristics of the European U‐BIOPRED adult severe asthma cohort. Eur Respir J. 2015;46(5):1308‐1321. doi:10.1183/13993003.00779-2015 Epub 2015 Sep 10 26357963

[29] Nair P , Surette MG , Virchow JC . Neutrophilic asthma: misconception or misnomer? Lancet Respir Med. 2021;9(5):441‐443. doi:10.1016/S2213-600(21)00023-0 Epub 2021 Feb 9 33577751

[30] Jabeen MF , Sanderson ND , Foster D , et al. Identifying bacterial airways infection in stable severe asthma using Oxford nanopore sequencing technologies. Microbiol Spectr. 2022;10(2):e0227921. doi:10.1128/spectrum.02279-21 Epub 2022 Mar 24 35323032 PMC9045196

[31] Abdel‐Aziz MI , Vijverberg SJH , Neerincx AH , et al. A multi‐omics approach to delineate sputum microbiome‐associated asthma inflammatory phenotypes. Eur Respir J. 2022;59(1):2102603. doi:10.1183/13993003.02603-2021 Print 2022 Jan 34824056

[32] Starkey MR , Plank MW , Casolari P , et al. IL‐22 and its receptors are increased in human and experimental COPD and contribute to pathogenesis. Eur Respir J. 2019;54(1). (pii):13993003.00174‐2018. doi:10.1183/.00174-2018 Print 2019 Jul PMC8132110 31196943

[33] McAleer JP , Kolls JK . Directing traffic: IL‐17 and IL‐22 coordinate pulmonary immune defense. Immunol Rev. 2014;260(1):129‐144. 24942687 10.1111/imr.12183 PMC4066195

[34] Tamasauskiene L , Sitkauskiene B . Interleukin‐22 in allergic airway diseases: a systematic review. J Interferon Cytokine Res. 2020;40(3):125‐130. doi:10.1089/jir.2019.0094 Epub 2019 Dec 31 31895598

[35] Bullone M , Carriero V , Bertolini F , et al. Elevated serum IgE, oral corticosteroid dependence and IL‐17/22 expression in highly neutrophilic asthma. Eur Respir J. 2019;54(5):1900068. doi:10.1183/13993003.00068-2019 Print 2019 Nov 31439682

[36] Pennino D , Bhavsar PK , Effner R , et al. IL‐22 suppresses IFN‐γ‐mediated lung inflammation in asthmatic patients. J Allergy Clin Immunol. 2013;131(2):562‐570. doi:10.1016/j.jaci.2012.09.036 Epub Nov 19 23174657

[37] Yang D , Li Y , Liu T , et al. IL‐1β promotes IL‐17A production of ILC3s to aggravate neutrophilic airway inflammation in mice. Immunology. 2023;29(10):13644. 10.1111/imm.13644 36988516

[38] Jonckheere AC , Seys SF , Steelant B , et al. Innate lymphoid cells are required to induce airway hyperreactivity in a murine neutrophilic asthma model. Front Immunol. 2022;13:849155. eCollection 2022. doi:10.3389/fimmu.2022.849155 35371094 PMC8965562

[39] Lamb D , De Sousa D , Quast K , et al. RORγt inhibitors block both IL‐17 and IL‐22 conferring a potential advantage over anti‐IL‐17 alone to treat severe asthma. Respir Res. 2021;22(1):158. doi:10.1186/s12931-021-01743-7 34022896 PMC8141258

[40] Ito T , Hirose K , Nakajima H . Bidirectional roles of IL‐22 in the pathogenesis of allergic airway inflammation. Allergol Int. 2019;68(1):4‐8. doi:10.1016/j.alit.2018.10.002 Epub Nov 10 30424940

[41] Wang J , Gao S , Zhang J , Li C , Li H , Lin J . Interleukin‐22 attenuates allergic airway inflammation in ovalbumin‐induced asthma mouse model. BMC Pulm Med. 2021;21(1):385. doi:10.1186/s12890-021-01698-x 34836520 PMC8620641

[42] Pérez‐Cruz M , Koné B , Porte R , et al. The toll‐like receptor 5 agonist flagellin prevents non‐typeable haemophilus influenzae‐induced infection in cigarette smoke‐exposed mice. PLoS One. 2021;16(3):e0236216. doi:10.1371/journal.pone eCollection 2021 33784296 PMC8009382

[43] Yan Z , Chen B , Yang Y , et al. Multi‐omics analyses of airway host‐microbe interactions in chronic obstructive pulmonary disease identify potential therapeutic interventions. Nat Microbiol. 2022;7(9):1361‐1375. doi:10.1038/s41564-022-01196-8 Epub 2022 Aug 22 35995842

[44] Gibson PG , Yang IA , Upham JW , et al. Effect of azithromycin on asthma exacerbations and quality of life in adults with persistent uncontrolled asthma (AMAZES): a randomised, double‐blind, placebo‐controlled trial. Lancet. 2017;390(10095):659‐668. 28687413 10.1016/S0140-6736(17)31281-3

[45] Yue M , Tao S , Gaietto K , Chen W . Omics approaches in asthma research: challenges and opportunities. Chin Med J Pulmonary and Critical Care Medicine. 2024. 10.1016/j.pccm.2024.02.002 PMC11332849 39170962

[46] Gautam Y , Johansson E , Mersha TB . Multi‐omics profiling approach to asthma: an evolving paradigm. J Personalized Medicine. 2022;12(1):66. 10.3390/jpm12010066 PMC8778153 35055381

[47] Li CX , Wheelock CE , Skold CM , Wheelock AM . Integration of multi‐omics datasets enables molecular classification of COPD. Eur Respir J. 2018;51(5). (pii):13993003.01930‐2017. doi:10.1183/.01930-2017 Print 2018 May 29545283

[48] Niazi SK . A critical analysis of the FDA's Omics‐Driven pharmacodynamic biomarkers to establish biosimilarity. Pharmaceuticals (Basel). 2023;16(11). 10.3390/ph16111556 PMC10675618 38004421

[49] Kermani NZ , Pavlidis S , Xie J , et al. Instability of sputum molecular phenotypes in U‐BIOPRED severe asthma. Eur Respir J. 2021;57(2):2001836. doi:10.1183/13993003.01836-2020 Print 2021 Feb 33008937 PMC7859503

[50] Zounemat Kermani N , Saqi M , Agapow P , et al. Type 2‐low asthma phenotypes by integration of sputum transcriptomics and serum proteomics. Allergy. 2021;76(1):380‐383. doi:10.1111/all.14573 Epub 2020 Sep 16 32865817

