# Supplementary material for: Endotypes of severe neutrophilic and eosinophilic asthma from multi‐omics integration of U‐BIOPRED sputum samples
Source: Clin Transl Med. 2024 Jul 28;14(7):e1771. doi: 10.1002/ctm2.1771 (PMC11283589; doi:10.1002/ctm2.1771)
Supplement: Supplementary file 1 — Supporting Information [file CTM2-14-e1771-s003.docx]

**Endotypes of severe neutrophilic and eosinophilic asthma from multi-omics integration of**

**U-BIOPRED sputum samples**

Nazanin Zounemat Kermani^1,2^, Chuan-Xing Li^3^, Ali Versi^1^, Yusef Badi^1^, Kai Sun^2^, Mahmoud I Abdel-Aziz^4^, Martina Bonatti^3^, Anke-Hilse Maitland-van der Zee^4^, Ratko Djukanovic^5^, Åsa Wheelock^3^, , Sven-Erik Dahlen^3^, Peter Howarth^5^, Yike Guo^2^, Kian Fan Chung^1, 2^, Ian M. Adcock^1, 2^ on behalf of U-BIOPRED Project Team

^1^National Heart and Lung Institute, Imperial College London, London, UK

^2^Data Science Institute, Imperial College, London, UK

^3^Institute of Environmental Medicine, Centre for Allergy Research, Karolinska Institute, Stockholm, Sweden

^4^Department of Pulmonology, Amsterdam UMC, University of Amsterdam, Amsterdam, The Netherlands

^5^NIHR Southampton Respiratory Biomedical Research Unit and Clinical and Experimental Sciences, Southampton, UK

**Supplementary material**

Supplementary Figures S1-S12

Supplementary Tables S1-S11

**Supplementary Table S1**. **Demographic features of subjects used in cluster analysis.**

|  | In | Out | fdr In versus Out |
| --- | --- | --- | --- |
| n | 72 | 437 |  |
| Age (n) | 52 (44.75-58.25, 72) | 52 (41-61, 437) | 0.888 |
| Gender (female, n) | 40 (56%, 72) | 265 (61%, 437) | 0.437 |
| BMI (n) | 26.42 (23.45-32.18, 72) | 27.58 (24.21-32.78, 437) | 0.229 |
| Diagnosis (MMA, n) | 15 (21%, 72) | 73 (17%, 437) | 0.2 |
| No. exac. (per year, n) | 1.5 (0-3, 72) | 2 (0-3, 436) | 0.561 |
| no. of severe exac per year | 1 (0-2, 72) | 1 (0-2, 436) | 0.938 |
| FEV1 (% predicted, n) | 70.16 (51.7-85.31, 72) | 71.23 (54.42-89.04, 433) | 0.403 |
| OCS use (%, n) | 48 (68%, 71) | 286 (66%, 433) | 0.891 |
| OCS (normalised dose mg, n) | 10 (5.31-15, 30) | 15 (10-25, 178) | **0.00646** |
| ICS use (at least twice a day, n) | 41 (57%, 72) | 17 0(39%, 437) | 0.075 |
| LABA_use (at least twice a day, n) | 17 (24%, 72) | 97 (22%, 437) | 0.799 |
| ACQ (avg 1-5, n) | 2 (0.95-3, 68) | 2 (1-3, 424) | 0.731 |
| AQLQ (average, n) | 4.78 (3.61-6, 70) | 4.75 (3.72-5.72, 428) | 0.64 |
| Total HADS (n) | 9 (3-18, 69) | 11 (5-17, 428) | 0.403 |
| Total SNOT (n) | 26 (13-36, 67) | 28 (15-42, 429) | 0.113 |
| Smoking status (current smoker, n) | 6 (8%, 72) | 36 (8%, 437) | 0.111 |
| NO (standard flow rate, n | 28 (18-54, 69) | 25 (15-46.25, 412) | 0.291 |
| Ige level (IU/ml, n) | 103.5 (45.62-245.75, 68) | 120 (48.75-324.75, 428) | 0.603 |
| Blood eos (cells/μl, n) | 0.24 (0.12-0.42, 70) | 0.2 (0.1-0.4, 426) | 0.15 |
| Blood neu (cells/μl, n) | 4.2 (3.29-6.28, 70) | 4.66 (3.5-6.3, 426) | 0.28 |
| Sputum neu (%, n) | 54.63 (29.66-74.37, 72) | 52.68 (32.7-71.03, 152) | 0.892 |
| Sputum eos (%, n) | 2.18 (0.19-16.34, 72) | 2.42 (0.59-8.64, 152) | 0.908 |
| Sputum lymphocyte (%, n) | 1.1 (0.62-1.97, 72) | 0.92 (0.39-1.78, 152) | 0.207 |
| Sputum macrophage (%, n) | 27.43 (14.44-50.17, 72) | 36.75 (14.68-51.09, 152) | 0.287 |
| Nasal polyps diagnosed (n, %) | 2 (3%, 72) | 31 (7%, 437) | 0.563 |
| Eczema diagnosed (n, %) | 2 (3%, 72) | 31 (7%, 437) | 0.208 |
| Allergic rhinitis diagnosed (n, %) | 2 (3%, 72) | 31 (7%, 437) | **0.03** |
| GERD diagnosed (n, %) | 2 (3%, 72) | 31 (7%, 437) | 0.361 |
| Hay fever diagnosed (n, %) | 2 (3%, 72) | 31 (7%, 437) | 0.15 |
| Eosinophils sputum (cell count, n) | 11 (1-85, 72) | 12 (3-43, 152) | 0.996 |
| Lymphocytes sputum (cell count, n) | 6 (3-10,72) | 4.5 (2-10, 152) | 0.129 |
| Macrophages sputum (cell count, n) | 148.5(77.75-268.25,72) | 181.5 (78.75-256.25, 152) | 0.7 |
| Neutrophils sputum (cell count, n) | 286 (154-384, 72) | 259 (161.5-380.25, 152) | 0.611 |
| hCRP (mg/L, n) | 1.55 (0.66-4.55, 70) | 1.9 (0.82-4.5, 429) | 0.383 |

**Supplementary Table S2**. **Number of asthmatics and features of each of 6 omics platforms**

|  | Number of asthmatics | Number of features | *Omics* technology |
| --- | --- | --- | --- |
| Transcriptomics | 72 | 20223 | Microarray |
| Transcriptomics (ES) | 72 | 1471 | GSVA + microarray |
| Proteomics | 72 | 1051 | somaSCAN |
| Proteomics | 72 | 232 | Shotgun proteomics |
| Microbiome | 72 | 1727 | 16S sequencing |
| Metagenomics | 72 | 197 | Shotgun sequencing |

**Supplementary Table S3. Data combinations used for clustering.**

| Data combination | Transcriptomics | | Proteomics | | Microbiome | |
| --- | --- | --- | --- | --- | --- | --- |
|  | Microarray | GSVA + microarray | somaSCAN | LC-MS/MS | 16S | metagenomics |
| All data | 1 | 1 | 1 | 1 | 1 | 1 |
| Proteomics and Microbiome |  |  | 1 | 1 | 1 | 1 |
| Transcriptomics and Microbiome | 1 | 1 |  |  | 1 | 1 |
| Transcriptomics and Proteomics | 1 | 1 | 1 | 1 |  |  |
| Proteomics |  |  | 1 | 1 |  |  |
| Microbiome |  |  |  |  | 1 | 1 |

Data were sorted into 3 types, i.e., transcriptomics, proteomics, and microbiome. Each of these data types includes 2 datasets, shown on the second row. 3^rd^ to 8^th^ rows show combinations of the data. 1 stands for the inclusion of the datasets in the data combination. The final column describes the data combinations, e.g., 111111 means ‘All data’ were included.

**Supplementary Table S4. Characteristics of OAC2 Membership: Comparing Neutrophilic to Mixed Granulocytic phenotypes.**

|  | **mixed** | **neutrophilic** | **Comparison p value** |
| --- | --- | --- | --- |
| **Subjects** | 6 | 5 | mixed neutrophilic |
| **Age (years)** | 56.5(48.5-57.75,6) | 42(32-63,5) | 0.401 |
| **Age of onset (years)** | 37.5(23-52,6) | 17(11-19,5) | 0.155 |
| **Female** | 5(83%,6) | 4(80%,5) | 1 |
| **BMI** | 25.46(24.56-29.6,6) | 29.86(23.67-32.8,5) | 0.62 |
| **Smoker** | 4(67%,6) | 3(60%,5) | 1 |
| **Nasal polyps (yes)** | 3(60%,5) | 2(40%,5) | 1 |
| **Allergic rhinitis** | 3(60%,5) | 3(75%,4) | 1 |
| **Eczema** | 2(40%,5) | 4(80%,5) | 0.539 |
| **Severe asthma** | 6(100%,6) | 4(80%,5) | 0.47 |
| **Oral corticosteroid use** | 4(67%,6) | 1(20%,5) | 0.244 |
| **Atopy (+)** | 5(83%,6) | 3(60%,5) | 0.541 |
| **Exacerbations previous year** | 2(1.25-2,6) | 2(1-3,5) | 0.85 |
| **FEV1 (L)** | 1.35(0.78-1.8,6) | 1.61(1.46-2.19,5) | 0.391 |
| **Total IgE (IU/mL)** | 150(69-186,6) | 243(162-740,5) | 0.429 |
| **Blood leukocytes ×10^3^ μL^−1^** | 8.3(7.53-9.47,6) | 9.9(9.3-10.7,5) | 0.512 |
| **Blood eosinophils** **×10^3^ μL^−1^** | 0.38(0.32-0.47,6) | 0.11(0.1-0.3,5) | 0.079 |
| **Blood neutrophils** **×10^3^ μL^−1^** | 5.58(4.44-6.23,6) | 6.53(4.8-7.1,5) | 0.756 |
| **Sputum eosinophils %** | 5.57(2.6625-13.9,6) | 0.19(0.18-0.19,5) | 0.008 |
| **Sputum neutrophils %** | 88.82(80.39-93.36,6) | 91.4(91.13-94.69,5) | 0.267 |
| **Sputum Macrophages %** | 3.88(3.01-4.41,6) | 7.85(4.13-8.3,5) | 0.14 |
| **FeNO (ppb)** | 54(26-130,5) | 16.5(13-22,5) | 0.095 |
| **Serum periostin (ng/mL)** | 63.51(45.8-70.18,5) | 49.85(47.79-49.92,5) | 0.548 |
| **CRP (mg/L)** | 3(2.1-26.4,6) | 6.4(4.8-7.8,5) | 0.784 |
| **Combined Atopy Regional Aeroallergens** | 5(83%,6) | 2(40%,5) | 0.235 |
| **History Pneumonia (+)** | 1(17%,6) | 0(0%,5) | 1 |

**Supplementary Table S5. Enriched biological terms and protein features associated with SA compared to MMA in OAC1. The table displays Gene Ontology (GO) processes, functions, and cellular components, UniProt keywords, and SMART protein domains that show significant enrichment in severe asthma. Observed gene counts, background gene counts, enrichment strength, FDR (false discovery rates), and corresponding proteins within the network are provided for each term or feature.**

| **category** | **term ID** | **term description** | **observed gene count** | **background gene count** | **FDR** | **matching proteins in your network (labels)** |
| --- | --- | --- | --- | --- | --- | --- |
| GO Process | GO:0006959 | Humoral immune response | 6 | 268 | 0.00076 | CFP,CCL21,C9,TNFRSF21,RARRES2,KNG1 |
| GO Process | GO:0007162 | Negative regulation of cell adhesion | 5 | 289 | 0.0191 | CCL21,TNFRSF21,EFNA5,EPHB2,KNG1 |
| GO Process | GO:0009605 | Response to external stimulus | 10 | 2355 | 0.0191 | RETN,CFP,CCL21,C9,EFNA5,EPHB2,UNC5C,RARRES2,ADAM9,KNG1 |
| GO Function | GO:0005102 | Signalling receptor binding | 8 | 1499 | 0.0416 | RETN,CCL21,EFNA5,EPHB2,RARRES2,ADAM9,MIA,KNG1 |
| GO Component | GO:0005576 | Extracellular region | 12 | 4175 | 0.0092 | PRSS22,RETN,CFP,CCL21,TIMP2,C9,COL18A1,EPHB2,RARRES2,ADAM9,MIA,KNG1 |
| GO Component | GO:0005615 | Extracellular space | 11 | 3247 | 0.0092 | PRSS22,RETN,CFP,CCL21,TIMP2,C9,COL18A1,RARRES2,ADAM9,MIA,KNG1 |
| GO Component | GO:0034774 | Secretory granule lumen | 5 | 321 | 0.0092 | RETN,CFP,TIMP2,RARRES2,KNG1 |
| GO Component | GO:0035580 | Specific granule lumen | 3 | 62 | 0.0092 | RETN,CFP,TIMP2 |
| GO Component | GO:0062023 | Collagen-containing extracellular matrix | 5 | 407 | 0.0092 | TIMP2,EFNA5,COL18A1,RARRES2,KNG1 |
| GO Component | GO:0005788 | Endoplasmic reticulum lumen | 4 | 312 | 0.0207 | CFP,COL18A1,TXNDC12,KNG1 |
| GO Component | GO:0031233 | Intrinsic component of external side of plasma membrane | 2 | 24 | 0.0369 | EFNA5,ADAM9 |
| COMPARTMENTS | GOCC:0005576 | Extracellular region | 12 | 2079 | 5.48E-06 | PRSS22,RETN,CFP,CCL21,TIMP2,C9,COL18A1,EPHB2,RARRES2,ADAM9,MIA,KNG1 |
| COMPARTMENTS | GOCC:0005615 | Extracellular space | 8 | 1027 | 0.00057 | PRSS22,RETN,CFP,C9,COL18A1,RARRES2,ADAM9,KNG1 |
| COMPARTMENTS | GOCC:0034774 | Secretory granule lumen | 5 | 241 | 0.00087 | RETN,CFP,TIMP2,RARRES2,KNG1 |
| COMPARTMENTS | GOCC:0035580 | Specific granule lumen | 3 | 62 | 0.0071 | RETN,CFP,TIMP2 |
| UniProt Keywords | KW-0732 | Signal | 16 | 3277 | 2.39E-10 | PRSS22,RETN,CFP,CCL21,TIMP2,C9,TNFRSF21,EFNA5,COL18A1,TXNDC12,EPHB2,UNC5C,RARRES2,ADAM9,MIA,KNG1 |
| UniProt Keywords | KW-1015 | Disulfide bond | 16 | 3338 | 2.39E-10 | PRSS22,RETN,CFP,CCL21,TIMP2,C9,TNFRSF21,EFNA5,COL18A1,TXNDC12,EPHB2,UNC5C,RARRES2,ADAM9,MIA,KNG1 |
| UniProt Keywords | KW-0964 | Secreted | 11 | 1839 | 3.03E-06 | PRSS22,RETN,CFP,CCL21,TIMP2,C9,COL18A1,RARRES2,ADAM9,MIA,KNG1 |
| UniProt Keywords | KW-0179 | Complement alternate pathway | 2 | 12 | 0.0094 | CFP,C9 |
| UniProt Keywords | KW-0395 | Inflammatory response | 3 | 163 | 0.0407 | CCL21,RARRES2,KNG1 |
| SMART | SM00209 | Thrombospondin type 1 repeats | 3 | 64 | 0.0167 | CFP,C9,UNC5C |

**Supplementary Table S6. Differentially enriched pathways in 5 omics-associated clusters (OAC1 to 5) compared to healthy volunteers (HV)**

| **Group comparison** | **Pathway** |
| --- | --- |
| **HV-OAC1** | None |
| **HV-OAC2** | Calcium-dependent regulation of normal and asthmatic smooth muscle contraction |
|  | Cell adhesion; Plasmin signaling |
|  | Cytoskeleton remodelling; Keratin filaments |
|  | Eosinophil adhesion and transendothelial migration in asthma |
|  | Eosinophil granule protein release in asthma |
|  | Immune response LPS-induced platelet activation |
|  | Immune response MIF - the neuroendocrine-macrophage connector |
|  | Inhibition of neutrophil migration by proresolving lipid mediators in COPD |
|  | Platelet activation as a result of endothelial dysfunction after stenting |
|  | Platelet activation during ADAM-TS13-deficient thrombotic microangiopathy development |
|  | Proteases and EGFR-induced mucin synthesis in normal and asthmatic epithelium |
|  | Role of integrins in eosinophil degranulation in asthma |
|  | Role of platelets in allograft rejection |
|  | Role of platelets in the initiation of in-stent restenosis |
|  | Stem cells; FGF signaling in pancreatic and hepatic differentiation of embryonic stem cells |
| **HV-OAC3** | Blood coagulation; Platelet; microparticle generation |
| **HV-OAC4** | None |
| **HV-OAC5** | None |

**Supplementary Table S7. The 5 differentially enriched (up- and down-regulated) pathways with highest and lowest median fold change between healthy volunteers (HV) and asthmatics clusters (OAC1-5)**

| **Dataset** | **Pathway** | **group comparison** | | **Median Fold change** | **P value** | **Q value** |
| --- | --- | --- | --- | --- | --- | --- |
| **shotgun** | Cytoskeleton remodeling;_Keratin filaments | HV | OAC1 | -20.94 | 6.96E-03 | 4.18E-02 |
| **shotgun** | Stem cells;_Schema: FGF signaling in embryonic stem cell self-renewal and differentiation | HV | OAC1 | -10.4 | 1.60E-04 | 3.37E-03 |
| **somaScan** | Role of iNKT and B cells in T cell recruitment in allergic contact dermatitis | HV | OAC1 | -4.64 | 2.79E-03 | 2.24E-02 |
| **transcriptomics** | Phospholipid metabolism p.3 | HV | OAC1 | -4.1 | 2.47E-03 | 2.06E-02 |
| **shotgun** | Stem cells_Direct reprogramming of cardiac fibroblasts into cardiomyocytes | HV | OAC1 | -3.65 | 5.41E-03 | 3.51E-02 |
| **transcriptomics** | Transcription_Negative regulation of HIF1A function | HV | OAC1 | 0.45 | 8.52E-03 | 4.80E-02 |
| **transcriptomics** | Development_Role of Activin A in cell differentiation and proliferation | HV | OAC1 | 0.48 | 3.20E-03 | 2.49E-02 |
| **transcriptomics** | Regulation of degradation of wtCFTR | HV | OAC1 | 0.52 | 7.98E-03 | 4.57E-02 |
| **somaScan** | Blood coagulation;_Platelet microparticle generation | HV | OAC1 | 2.24 | 4.12E-03 | 2.95E-02 |
| **somaScan** | Th2 cytokine- and TNF-alpha-induced inflammatory response in asthmatic airway fibroblasts | HV | OAC1 | 3.12 | 8.90E-03 | 4.94E-02 |
| **somaScan** | Chylomicron dyslipidemia in type 2 diabetes and metabolic syndrome X | HV | OAC2 | -19.27 | 2.57E-03 | 2.13E-02 |
| **somaScan** | Growth factors in regulation of oligodendrocyte precursor cells survival in multiple sclerosis | HV | OAC2 | -11.48 | 4.26E-03 | 3.01E-02 |
| **somaScan** | Influence of bone marrow cell environment on progression of multiple myeloma | HV | OAC2 | -6.65 | 3.52E-03 | 2.65E-02 |
| **somaScan** | Pro-oncogenic action of Androgen receptor in breast cancer | HV | OAC2 | -6.57 | 7.93E-03 | 4.55E-02 |
| **somaScan** | Defective macrophage-mediated bacterial phagocytosis in COPD | HV | OAC2 | -6.47 | 1.84E-03 | 1.70E-02 |
| **somaScan** | Signal transduction_PTMs in BAFF-induced canonical NF-κB signaling | HV | OAC2 | 0.31 | 3.74E-03 | 2.78E-02 |
| **somaScan** | Immune response_IL-9 signaling pathway | HV | OAC2 | 0.32 | 7.00E-03 | 4.20E-02 |
| **somaScan** | Apoptosis and survival_Cytoplasmic/mitochondrial transport of proapoptotic proteins Bid, Bmf and Bim | HV | OAC2 | 0.33 | 4.75E-03 | 3.22E-02 |
| **somaScan** | G protein-coupled receptors signaling in lung cancer | HV | OAC2 | 0.45 | 9.03E-03 | 4.96E-02 |
| **somaScan** | Th2 cytokine- and TNF-alpha-induced inflammatory response in asthmatic airway fibroblasts | HV | OAC2 | 77.84 | 6.97E-03 | 4.18E-02 |
| **somaScan** | Schema: Initiation of T cell recruitment in allergic contact dermatitis | HV | OAC3 | -33.27 | 6.78E-03 | 4.10E-02 |
| **shotgun** | Cell adhesion_Integrin-mediated cell adhesion and migration | HV | OAC3 | -14.78 | 3.87E-03 | 2.84E-02 |
| **shotgun** | Impaired inhibitory action of lipoxins on neutrophil migration in CF | HV | OAC3 | -14.78 | 3.87E-03 | 2.84E-02 |
| **transcriptomics** | Phospholipid metabolism p.3 | HV | OAC3 | -13.82 | 7.38E-03 | 4.36E-02 |
| **somaScan** | Proinflammatory mediators production and activation of basophils in asthma | HV | OAC3 | -13.4 | 3.54E-03 | 2.66E-02 |
| **somaScan** | T regulatory cells in asthma | HV | OAC3 | 0.4 | 8.24E-03 | 4.67E-02 |
| **somaScan** | Metabolic syndrome X (general schema) | HV | OAC3 | 18.08 | 4.83E-03 | 3.26E-02 |
| **transcriptomics** | Immune response_LPS-induced platelet activation | HV | OAC3 | 18.42 | 6.34E-03 | 3.94E-02 |
| **shotgun** | Development_Regulation of endothelial progenitor cell differentiation from adult stem cells | HV | OAC3 | 23.02 | 2.68E-03 | 2.19E-02 |
| **transcriptomics** | Development_Transcriptional regulation of megakaryopoiesis | HV | OAC3 | 62.24 | 6.86E-03 | 4.13E-02 |
| **transcriptomics** | Ubiquinone metabolism | HV | OAC4 | -56.33 | 8.48E-03 | 4.78E-02 |
| **transcriptomics** | Medium-chain saturated fatty acids synthesis | HV | OAC4 | -15.23 | 3.99E-03 | 2.88E-02 |
| **transcriptomics** | Propionate metabolism p.1 | HV | OAC4 | -11.25 | 6.54E-03 | 3.99E-02 |
| **transcriptomics** | Eosinophil granule protein release in asthma | HV | OAC4 | -10.28 | 1.70E-03 | 1.60E-02 |
| **somaScan** | Th2 cytokine- and TNF-alpha-induced inflammatory response in asthmatic airway fibroblasts | HV | OAC4 | -8.99 | 4.27E-03 | 3.01E-02 |
| **transcriptomics** | Environmental factors-induced inflammatory signaling in normal and asthmatic airway epithelium | HV | OAC4 | 12.7 | 3.43E-03 | 2.61E-02 |
| **transcriptomics** | Tricarbonic acid cycle | HV | OAC4 | 13.27 | 8.76E-04 | 1.01E-02 |
| **shotgun** | Cytoskeleton remodeling_Keratin filaments | HV | OAC4 | 17.24 | 7.90E-03 | 4.54E-02 |
| **transcriptomics** | Proline metabolism | HV | OAC4 | 26.01 | 1.77E-03 | 1.65E-02 |
| **transcriptomics** | GTP-XTP metabolism | HV | OAC4 | 38.08 | 1.78E-03 | 1.65E-02 |
| **transcriptomics** | Neurophysiological process_Netrin-1 in regulation of axon guidance | HV | OAC5 | -154.15 | 4.41E-03 | 3.07E-02 |
| **transcriptomics** | CHDI_Correlations from Replication data_Causal network (negative correlations) | HV | OAC5 | -11.01 | 1.73E-03 | 1.62E-02 |
| **somaScan** | Transport_Intracellular cholesterol transport | HV | OAC5 | -8.74 | 3.75E-03 | 2.78E-02 |
| **transcriptomics** | DNA damage_Role of Brca1 and Brca2 in DNA repair | HV | OAC5 | -8.71 | 5.26E-03 | 3.45E-02 |
| **somaScan** | Fenofibrate in treatment of type 2 diabetes and metabolic syndrome X | HV | OAC5 | -7.01 | 8.20E-03 | 4.66E-02 |
| **somaScan** | Development_Leptin signaling via JAK/STAT and MAPK cascades | HV | OAC5 | 0.53 | 9.19E-03 | 5.00E-02 |
| **somaScan** | Role of IL-8 in melanoma | HV | OAC5 | 0.53 | 9.19E-03 | 5.00E-02 |
| **transcriptomics** | FGF2 signaling in melanoma | HV | OAC5 | 3.41 | 4.37E-03 | 3.05E-02 |
| **somaScan** | Aberrant lipid trafficking and metabolism in age-related macular degeneration pathogenesis | HV | OAC5 | 9.61 | 6.94E-03 | 4.17E-02 |
| **transcriptomics** | Leucine, isoleucine and valine metabolism.p.2 | HV | OAC5 | 846.64 | 6.44E-03 | 3.96E-02 |

**Supplementary Table S8. The 5 differentially enriched pathways with the highest and lowest median fold change between each OAC and the remaining subjects**

| **Dataset** | **Pathway** | **group comparison** | **Median Fold change** | **P value** | **Q value** |
| --- | --- | --- | --- | --- | --- |
| **somaScan** | Some pathways of EMT in cancer cells | OAC1-Rest | -134.09 | 1.61E-02 | 4.92E-02 |
| **transcriptomics** | Membrane-bound ESR1: interaction with G-proteins signaling | OAC1-Rest | -102.8 | 1.24E-02 | 4.02E-02 |
| **transcriptomics** | HGF receptor (Met) and MSP receptor (RON) signaling pathways in SCLC | OAC1-Rest | -87.15 | 1.58E-02 | 4.83E-02 |
| **transcriptomics** | Angiotensin II Signaling in Cardiac Hypertrophy | OAC1-Rest | -63.84 | 3.19E-04 | 2.49E-03 |
| **transcriptomics** | Development_IGF-1 receptor signaling | OAC1-Rest | -54.12 | 1.60E-03 | 8.43E-03 |
| **transcriptomics** | Bromobenzene metabolism/Rodent version | OAC1-Rest | 18.68 | 8.39E-03 | 2.97E-02 |
| **transcriptomics** | HDL dyslipidemia in type 2 diabetes and metabolic syndrome X | OAC1-Rest | 28.59 | 1.10E-02 | 3.69E-02 |
| **transcriptomics** | Suppression of p53 signaling in multiple myeloma | OAC1-Rest | 32.09 | 3.85E-03 | 1.63E-02 |
| **transcriptomics** | Immune response_KLRK1 (NKG2D) signaling pathway | OAC1-Rest | 52.26 | 1.17E-02 | 3.83E-02 |
| **somaScan** | Apoptosis and survival_Beta-2 adrenergic receptor anti-apoptotic action | OAC1-Rest | 57.61 | 6.24E-03 | 2.37E-02 |
| **transcriptomics** | GTP-XTP metabolism | OAC2-Rest | -237944.22 | 1.35E-05 | 2.50E-04 |
| **transcriptomics** | Role of type 2 innate lymphoid cells in asthma | OAC2-Rest | -1511.56 | 1.30E-04 | 1.28E-03 |
| **transcriptomics** | IL-1 beta- and Endothelin-1-induced fibroblast/ myofibroblast migration and extracellular matrix production in asthmatic airways | OAC2-Rest | -614.13 | 3.25E-05 | 4.66E-04 |
| **somaScan** | VLDL, LDL dyslipidemia in type 2 diabetes and metabolic syndrome X | OAC2-Rest | -575.26 | 1.02E-02 | 3.45E-02 |
| **transcriptomics** | Immune response_Lysophosphatidic acid signaling via NF-κB | OAC2-Rest | -554.91 | 1.24E-04 | 1.23E-03 |
| **transcriptomics** | HBV mediates angiogenesis in HCC | OAC2-Rest | 274.27 | 1.73E-04 | 1.58E-03 |
| **somaScan** | Nicotine signaling in dopaminergic neurons, Pt. 2 - axon terminal | OAC2-Rest | 324.38 | 6.38E-04 | 4.15E-03 |
| **somaScan** | Platelet activation during ADAM-TS13-deficient thrombotic microangiopathy development | OAC2-Rest | 362.47 | 1.44E-02 | 4.50E-02 |
| **shotgun** | Inhibition of neutrophil migration by proresolving lipid mediators in COPD | OAC2-Rest | 782.19 | 6.75E-04 | 4.35E-03 |
| **somaScan** | Cell adhesion_Gap junctions | OAC2-Rest | 6272.57 | 3.38E-03 | 1.47E-02 |
| **transcriptomics** | Role of platelets in the initiation of in-stent restenosis | OAC3-Rest | -1887.29 | 3.78E-03 | 1.60E-02 |
| **transcriptomics** | DNA damage_DNA-damage-induced responses | OAC3-Rest | -52.81 | 1.65E-03 | 8.62E-03 |
| **transcriptomics** | Eosinophil survival in asthma | OAC3-Rest | -36.86 | 1.12E-02 | 3.72E-02 |
| **transcriptomics** | Development_Hedgehog signaling | OAC3-Rest | -32.77 | 8.14E-05 | 9.18E-04 |
| **transcriptomics** | Oxidative stress_NOX and DUOX families of NADPH oxidases | OAC3-Rest | -29.65 | 1.64E-02 | 4.98E-02 |
| **somaScan** | MAPK-mediated proliferation of normal and asthmatic smooth muscle cells | OAC3-Rest | 30.21 | 9.21E-03 | 3.20E-02 |
| **transcriptomics** | Lacto-series GSL Metabolism | OAC3-Rest | 49.7 | 2.94E-05 | 4.35E-04 |
| **shotgun** | Role of integrins in eosinophil degranulation in asthma | OAC3-Rest | 53.57 | 1.35E-02 | 4.29E-02 |
| **somaScan** | Chylomicron dyslipidemia in type 2 diabetes and metabolic syndrome X | OAC3-Rest | 224.13 | 3.58E-03 | 1.54E-02 |
| **transcriptomics** | WNT signaling in gastric cancer | OAC3-Rest | 427.72 | 4.40E-03 | 1.81E-02 |
| **transcriptomics** | Role of type 2 innate lymphoid cells in asthma | OAC4-Rest | -1473.54 | 3.73E-03 | 1.58E-02 |
| **transcriptomics** | IGF signaling in HCC | OAC4-Rest | -784.58 | 1.57E-02 | 4.80E-02 |
| **transcriptomics** | IgE- and MGF-induced Fyn-mediated activation of lung mast cells in asthma | OAC4-Rest | -438.03 | 4.79E-04 | 3.37E-03 |
| **transcriptomics** | Immune response_Lysophosphatidic acid signaling via NF-κB | OAC4-Rest | -418.66 | 3.06E-03 | 1.37E-02 |
| **transcriptomics** | Immune response_IL-17 signaling pathways | OAC4-Rest | -330.2 | 4.95E-03 | 1.99E-02 |
| **transcriptomics** | Vascular endothelial cell damage in SLE | OAC4-Rest | 190.44 | 2.22E-03 | 1.07E-02 |
| **transcriptomics** | Gamma-secretase regulation of osteogenesis | OAC4-Rest | 196.7 | 4.83E-05 | 6.21E-04 |
| **transcriptomics** | Role of Apo-2L(TNFSF10) in Prostate Cancer cell apoptosis | OAC4-Rest | 615.5 | 1.05E-03 | 6.09E-03 |
| **transcriptomics** | Immune response_Inhibitory PD-1 signaling in T cells | OAC4-Rest | 773.07 | 1.21E-02 | 3.95E-02 |
| **somaScan** | Signal transduction_PTMs in BAFF-induced canonical NF-κB signaling | OAC4-Rest | 892.89 | 1.21E-02 | 3.95E-02 |
| **somaScan** | Stem cells_Hypothetical role of microRNAs in fibrosis development after myocardial infarction | OAC5-Rest | -8.46E+13 | 1.60E-02 | 4.88E-02 |
| **somaScan** | Influence of smoking on activation of EGFR signaling in lung cancer cells | OAC5-Rest | -294 | 4.38E-03 | 1.80E-02 |
| **somaScan** | Development_Thromboxane A2 signaling pathway | OAC5-Rest | -176.57 | 3.04E-03 | 1.37E-02 |
| **somaScan** | Immune response_IFN-alpha/beta signaling via MAPKs | OAC5-Rest | -147.38 | 6.80E-03 | 2.54E-02 |
| **somaScan** | Regulation of lipid metabolism_Insulin signaling: generic cascades | OAC5-Rest | -140.71 | 1.13E-03 | 6.41E-03 |
| **somaScan** | Stimulation of gastric acid secretion in gastric cancer | OAC5-Rest | 206.84 | 1.33E-02 | 4.25E-02 |
| **shotgun** | Cytoskeleton remodeling_ESR1 action on cytoskeleton remodeling and cell migration | OAC5-Rest | 248.99 | 5.24E-04 | 3.61E-03 |
| **somaScan** | CHDI_Correlations from Discovery data_Causal network | OAC5-Rest | 274.8 | 1.46E-02 | 4.56E-02 |
| **somaScan** | Growth factors in regulation of oligodendrocyte precursor cells proliferation in multiple sclerosis | OAC5-Rest | 1159.9 | 1.50E-03 | 8.03E-03 |
| **somaScan** | Glucocorticoids-mediated inhibition of pro-constrictory and pro-inflammatory signaling in airway smooth muscle cells | OAC5-Rest | 770013.73 | 3.12E-03 | 1.39E-02 |

**Supplementary Table S9. Pathways repeatedly enriched in all groups’ comparison across OACs.**

| **Pathway** |
| --- |
| Muscle contraction - Relaxin signaling pathway |
| HBV-dependent NF-kB and PI3K/AKT pathways leading to HCC |
| Inhibition of apoptosis in gastric cancer |
| Stem cells - FGF2 signaling during embryonic stem cell differentiation |
| Cigarette smoke-mediated attenuation of antibacterial and antivirus immune response |
| Apoptotic pathways and resistance to apoptosis in lung cancer cells |
| Role of IL-8 in melanoma |
| Immune response - IL-33 signaling pathway |
| Role of integrins in eosinophil degranulation in asthma |
| Signal transduction - Soluble CXCL16 signaling |
| Apoptosis and survival - Caspase cascade |
| G-protein signalling - N-RAS regulation pathway |
| Development VEGF - family signaling |
| Apoptosis and survival - Role of IAP-proteins in apoptosis |
| Development NCAM1 - mediated neurite outgrowth, synapse assembly and neuronal survival |
| Apoptosis and survival - BAD phosphorylation |
| Cytoskeleton remodelling - Integrin outside-in signaling |
| Apoptosis and survival - Apoptotic TNF-family pathways |
| Cell cycle - ESR1 regulation of G1/S transition |
| Transport - Aldosterone-mediated regulation of ENaC sodium transport |
| Immune response - IL-3 signaling via ERK and PI3K |
| Apoptosis and survival - NO signaling in survival |
| Role of ER stress in obesity and type 2 diabetes |
| K-RAS signaling in lung cancer |
| Calcium-dependent regulation of normal and asthmatic smooth muscle contraction |
| IL-6 signaling pathway in lung cancer |
| Suppression of p53 signaling in multiple myeloma |
| Neutrophil resistance to apoptosis in COPD and proresolving impact of lipid mediators |
| Signal transduction - PTMs in BAFF-induced non-canonical NF-kB signaling |
| T regulatory cells in asthma |
| Role of epigenetic alterations in survival and migration of SCLC cells |
| Vascular endothelial cell damage in SLE |

**Supplementary Table S10. - Enrichment of neutrophil, TH1, TH17, ILC3 and Macrophage signatures and pathways across OAC2, OAC4 and healthy.**

|  | **Signature / pathway** | **OAC2** | **OAC4** | **HV** | **OAC2 - OAC4** | **OAC2 – HV** | **OAC4 -HV** |
| --- | --- | --- | --- | --- | --- | --- | --- |
| **Neutrophils** | IgE dependent production of pro inflammatory mediators by neutrophils in asthma | 0.2(0.2-0.3) | 0(-0.1-0.2) | -0.2(-0.3-0.1) | 0.022 | 6e-05 | 0.050 |
|  | Neutrophil | 0.6(0.4-0.6) | 0.2(0-0.4) | -0.3(-0.6-0.1) | 0.009 | 6e-05 | 0.015 |
|  | Neutrophil adhesion and transendothelial migration in asthma | 0.2(0.1-0.4) | 0(-0.1-0.3) | 0(-0.2-0.1) | ns | 0.007 | ns |
|  | Neutrophil chemotaxis in asthma | 0.2(0.1-0.3) | 0.1(0.1-0.2) | -0.2(-0.3-0) | ns | 1e-05 | 6e-04 |
|  | Neutrophil derived granule proteins and cytokines in asthma | 0.3(0.3-0.4) | 0(0-0.1) | -0.3(-0.3-0) | 1e-04 | 1e-07 | 0.010 |
|  | Polymorphonuclear neutrophils | 0.6(-0.3-0.7) | -0.6(-0.7--0.5) | -0.6(-0.7-0.4) | 0.0072 | 0.0400 | 0.5306 |
| **Neutrophil activation** | Positive regulation of neutrophil activation | 0.3(0-0.6) | 0.4(0-0.6) | -0.2(-0.4-0.2) | ns | 0.040 | 0.013 |
|  | Neutrophil activation | -0.1(-0.2-0) | 0.3(0.1-0.3) | 0.2(0-0.3) | 4e-05 | 0.008 | ns |
|  | Negative regulation of neutrophil activation | 0.4(0.1-0.6) | 0(-0.3-0.4) | -0.4(-0.6-0.2) | ns | 5e-05 | 0.008 |
|  | Regulation of neutrophil activation | 0.2(-0.1-0.4) | 0.2(-0.1-0.5) | -0.1(-0.3-0.2) | ns | Ns | 0.041 |
| **Neutrophil aggregation** | Neutrophil aggregation | 0.5(0.4-0.8) | 0.4(0.1-0.6) | 0(-0.3-0.7) | ns | Ns | ns |
|  | Regulation of neutrophil aggregation | -0.7(-0.9-0.7) | 0(0-0.1) | 0.5(0.1-0.7) | 4e-07 | 6e-08 | 0.022 |
| **Neutrophil apoptosis** | Negative regulation of neutrophil apoptotic process | 0.1(0-0.3) | 0.3(0.1-0.5) | -0.4(-0.5-0) | ns | 0.016 | 5e-04 |
|  | Neutrophil apoptotic process | 0.5(0.1-0.7) | 0.1(-0.2-0.5) | 0(-0.3-0.2) | ns | 0.023 | ns |
|  | Positive regulation of neutrophil apoptotic process | 0(-0.1-0.2) | 0.4(0.2-0.5) | 0(-0.4-0.2) | ns | Ns | 0.005 |
|  | Regulation of neutrophil apoptotic process | 0.1(0-0.3) | 0.3(0.2-0.5) | -0.2(-0.5-0.1) | 0.022 | Ns | 0.001 |
| **Neutrophil bacterial killing** | Neutrophil mediated killing of gram negative bacterium | 0.2(0-0.4) | -0.1(-0.3-0.1) | 0(-0.4-0.3) | 0.008 | Ns | ns |
|  | Positive regulation of neutrophil mediated killing of gram negative bacterium | 0.6(0.2-0.6) | -0.1(-0.6-0.7) | -0.2(-0.5-0.5) | ns | 0.045 | ns |
|  | Regulation of neutrophil mediated killing of gram negative bacterium | 0.3(0.2-0.8) | -0.2(-0.5-0.4) | 0(-0.4-0.5) | ns | 0.042 | ns |
| **Neutrophil bactericidal activity** | Neutrophil mediated killing of bacterium | 0.1(0-0.5) | -0.3(-0.3-0) | -0.1(-0.3-0) | 0.002 | 0.016 | ns |
|  | Regulation of neutrophil mediated killing of bacterium | 0.3(0.2-0.8) | -0.2(-0.5-0.4) | 0(-0.4-0.5) | ns | 0.042 | ns |
|  | Neutrophil mediated killing of gram positive bacterium | 0.3(-0.8-0.6) | -0.4(-0.8-0.1) | -0.1(-0.4-0.4) | ns | Ns | ns |
|  | Positive regulation of neutrophil mediated killing of bacterium | 0.6(0.2-0.6) | -0.1(-0.6-0.7) | -0.2(-0.5-0.5) | ns | 0.045 | ns |
| **Neutrophil chemotaxis** | Neutrophil chemotaxis | 0.2(0.2-0.4) | 0.1(0.1-0.3) | -0.2(-0.3-0) | ns | 5e-05 | 1e-04 |
|  | Regulation of neutrophil chemotaxis | 0.1(0-0.2) | 0.1(0-0.3) | -0.1(-0.2-0) | ns | 0.017 | 0.012 |
|  | Positive regulation of neutrophil chemotaxis | 0.1(0-0.2) | 0.2(0.1-0.3) | -0.1(-0.2-0) | ns | 0.037 | 0.008 |
|  | Negative regulation of neutrophil chemotaxis | 0.1(-0.2-0.5) | -0.5(-0.7-0.3) | 0(-0.4-0.4) | 0.020 | Ns | ns |
| **Neutrophil clearance** | Neutrophil clearance | -0.4(-0.6-0.2) | 0.4(0.1-0.5) | 0.2(0-0.5) | 2e-04 | 9e-05 | ns |
| **Neutrophil cytotoxicity** | Positive regulation of neutrophil mediated cytotoxicity | 0.4(0.1-0.6) | 0(-0.4-0.2) | -0.1(-0.4-0) | 0.022 | 4e-04 | ns |
|  | Neutrophil mediated cytotoxicity | 0.1(0-0.5) | -0.3(-0.3-0) | -0.1(-0.3-0) | 0.002 | 0.016 | ns |
|  | Regulation of neutrophil mediated cytotoxicity | 0.3(0.2-0.5) | -0.1(-0.2-0.1) | -0.3(-0.4-0) | 0.038 | 1e-04 | ns |
| **Neutrophil degranulation** | Negative regulation of neutrophil degranulation | -0.3(-0.6-0.3) | -0.3(-0.6-0.2) | -0.1(-0.6-0.3) | ns | Ns | ns |
|  | Positive regulation of neutrophil degranulation | 0.3(0-0.6) | 0.4(0-0.6) | -0.2(-0.4-0.2) | ns | 0.040 | 0.013 |
|  | Neutrophil degranulation | -0.1(-0.2-0) | 0.3(0.1-0.3) | 0.2(0-0.3) | 1e-04 | 0.009 | ns |
|  | Regulation of neutrophil degranulation | -0.1(-0.3-0.3) | 0.3(-0.2-0.5) | 0(-0.2-0.2) | ns | Ns | ns |
| **Neutrophil differentiation** | Positive regulation of neutrophil differentiation | 0.1(0-0.7) | 0.3(0-0.5) | 0.2(-0.1-0.4) | ns | Ns | ns |
|  | Neutrophil differentiation | 0.1(-0.1-0.2) | 0.1(-0.4-0.2) | -0.3(-0.4-0) | ns | 0.005 | ns |
|  | Negative regulation of neutrophil differentiation | 0.8(0.6-0.9) | -0.4(-0.6-0.2) | -0.2(-0.5-0.1) | 4e-04 | 3e-04 | ns |
|  | Regulation of neutrophil differentiation | 0.6(0.4-0.7) | 0(-0.4-0.3) | 0(-0.3-0.2) | 0.006 | 0.002 | ns |
| **Neutrophil extravasation** | Neutrophil extravasation | 0.1(0-0.5) | 0.2(-0.1-0.5) | 0.2(0-0.4) | ns | Ns | ns |
|  | Regulation of neutrophil extravasation | 0.3(0.2-0.4) | 0.1(0-0.2) | -0.1(-0.2-0) | 0.029 | 1e-05 | 0.017 |
|  | Positive regulation of neutrophil extravasation | 0.3(0.1-0.4) | 0.2(0-0.3) | -0.1(-0.2-0.1) | ns | 1e-04 | 0.013 |
| **Neutrophil fungal killing** | Positive regulation of neutrophil mediated killing of fungus | 0.4(0.3-0.5) | 0(-0.5-0.4) | -0.1(-0.5-0.1) | ns | 0.008 | ns |
|  | Neutrophil mediated killing of fungus | 0.3(-0.4-0.7) | -0.1(-0.8-0.3) | -0.4(-0.5-0) | ns | Ns | ns |
|  | Regulation of neutrophilmediated killing of fungus | 0.4(0.3-0.5) | 0(-0.5-0.4) | -0.1(-0.5-0.1) | ns | 0.008 | ns |
| **Neutrophil homeostasis** | Neutrophil homeostasis | 0(-0.2-0.2) | 0.1(-0.1-0.2) | 0(-0.1-0.1) | ns | Ns | ns |
| **Neutrophil immune response** | Neutrophil activation involved in immune response | -0.1(-0.2-0) | 0.3(0.1-0.3) | 0.2(0-0.3) | 3e-05 | 0.006 | ns |
| **Neutrophil immunity** | Neutrophil mediated immunity | -0.1(-0.2-0.1) | 0.3(0.1-0.3) | 0.2(0-0.3) | 1e-04 | 0.012 | ns |
| **Neutrophil migration** | Negative regulation of neutrophil migration | -0.3(-0.6-0) | -0.2(-0.3-0.3) | 0.2(0-0.6) | ns | 0.031 | ns |
|  | Neutrophil migration | 0.3(0.1-0.3) | 0.2(0.1-0.3) | -0.1(-0.2-0) | ns | 1e-04 | 1e-04 |
|  | Positive regulation of neutrophil migration | 0.1(0-0.2) | 0.2(0.1-0.3) | -0.1(-0.2-0) | ns | 0.004 | 0.002 |
|  | Regulation of neutrophil migration | 0.2(0-0.2) | 0.1(0.1-0.3) | -0.1(-0.2-0) | ns | 0.010 | 0.004 |
| **Neutrophil symbiont killing** | Positive regulation of neutrophil mediated killing of symbiont cell | 0.4(0.1-0.6) | 0(-0.4-0.2) | -0.1(-0.4-0) | 0.022 | 4e-04 | Ns |
|  | Regulation of neutrophil mediated killing of symbiont cell | 0.4(0.1-0.6) | 0(-0.2-0.1) | -0.1(-0.4-0.1) | 0.037 | 7e-04 | Ns |
|  | Neutrophil mediated killing of symbiont cell | 0.1(0-0.5) | -0.3(-0.3-0) | -0.1(-0.3-0) | 0.002 | 0.016 | Ns |
| **Neutrophils aged VS activated Signature** | Neutrophils aged VS activated up nature15367 | -0.1(-0.2-0.1) | 0.2(0.1-0.3) | 0.1(-0.2-0.3) | 0.002 | Ns | Ns |
| **NETosis related genes.** | Netosis | 0.4(0.3-0.5) | -0.2(-0.3-0.3) | -0.3(-0.5-0.4) | 0.0060 | 0.011 | Ns |
| **Th1, Th17** | Th17 specific | 0.5(0.1-0.6) | 0.5(0.3-0.8) | -0.4(-0.5-0.1) | ns | 3e-04 | 4e-04 |
|  | Th1 specific | 0.3(-0.1-0.6) | 0.4(0.3-0.6) | -0.1(-0.4-0.2) | ns | 0.046 | 0.004 |
|  | Th17 cells in CF | 0.1(-0.1-0.2) | 0.2(0.2-0.4) | 0(-0.2-0.2) | 0.049 | Ns | 0.003 |
|  | Th17 cells in CF mouse model. | 0.1(0-0.2) | 0.2(0.1-0.3) | 0(-0.3-0.2) | 0.049 | Ns | 0.005 |
|  | Immune response- Th1 and Th2 cell differentiation | 0(-0.2-0.2) | 0.3(0.1-0.3) | -0.1(-0.2-0.1) | ns | Ns | 0.006 |
|  | Immune response - Th17 cell differentiation | 0.1(-0.1-0.1) | 0.3(0.1-0.3) | 0(-0.2-0.2) | 0.040 | Ns | 0.041 |
|  | Impaired inhibition of Th17 cell differentiation by IFNbeta in multiple sclerosis | 0.3(0.2-0.4) | 0.3(0.2-0.4) | -0.2(-0.4-0.1) | ns | 0.002 | 0.006 |
|  | Immune response - Th17, Th22 and Th9 cell differentiation. | 0(0-0.2) | 0.2(0.1-0.3) | 0(-0.2-0.1) | 0.037 | Ns | 0.008 |
|  | Th1 and Th17 cells in an autoimmune mechanism of emphysema formation in smokers | -0.2(-0.4-0.1) | 0.3(0.1-0.4) | 0(0-0.2) | 6e-04 | Ns | 0.032 |
|  | Role of Th17 cells in asthma | 0.2(0.1-0.3) | 0(0-0.2) | -0.2(-0.3-0) | ns | 2e-04 | 0.010 |
|  | Proinflammatory cytokine production by Th17 cells in asthma | 0(-0.2-0.1) | 0.3(0.1-0.4) | 0(-0.2-0.1) | 9e-04 | Ns | 0.001 |
|  | Common mechanisms of Th17 cell migration | 0.1(0-0.2) | 0.1(0-0.2) | -0.1(-0.2-0) | ns | 0.001 | 4e-04 |
| **ILC3** | siLP ILC3 MM UP | -0.1(-0.2-0.1) | 0.2(0-0.3) | 0(-0.2-0.1) | 0.013 | Ns | 0.020 |
|  | ILC3 – up -PMID26878113 | 0(0-0.1) | 0(0-0.1) | -0.1(-0.1-0) | ns | Ns | 0.039 |
| **Macrophages** | Stimuli - Fatty acids (LA, OA) | -0.2(-0.4-0) | 0.2(0.2-0.3) | 0.2(0.1-0.3) | 2e-07 | 3e-04 | ns |
|  | Stimuli - Fatty acids (LA) | -0.3(-0.4-0.2) | 0.3(0.2-0.4) | 0.4(0.2-0.4) | 2e-07 | 1e-05 | ns |
|  | Stimuli - M1 (LPS, LPS + IFN-γ) | -0.3(-0.3-0.1) | 0.2(0.2-0.2) | 0.2(0-0.3) | 1e-06 | 2e-04 | ns |
|  | Stimuli - Fatty acids (OA, PA) | -0.2(-0.4-0.2) | 0.2(0.1-0.2) | 0.3(0-0.3) | 3e-07 | 5e-06 | ns |
|  | Stimuli - Fatty acids (PA, OA, LA) | -0.2(-0.3-0.1) | 0.3(0.2-0.3) | 0.3(0.1-0.3) | 6e-06 | 3e-04 | ns |
|  | Stimuli - Fatty acids (OA, LA, PA) | -0.4(-0.4-0.2) | 0.2(0.2-0.3) | 0.3(0.1-0.4) | 2e-07 | 2e-06 | ns |
|  | Stimuli - M1 (IFN-γ) | -0.1(-0.2-0.1) | 0.1(0-0.4) | 0.3(0.1-0.5) | ns | 0.045 | ns |
|  | Stimuli - M1 (IFN-γ, IFN-γ+ TNF-α) | 0.2(0.1-0.3) | 0.1(-0.1-0.4) | 0.1(-0.1-0.3) | ns | Ns | ns |
|  | Stimuli - M1 (IFN-γ + TNF-α, IFN-γ) | -0.1(-0.2-0) | 0.3(0.2-0.4) | 0.1(-0.1-0.2) | 5e-04 | Ns | 0.006 |
|  | Stimuli - M2 (IL-4, IL-10) | -0.5(-0.5-0.3) | 0.3(0.2-0.4) | 0.4(0.3-0.5) | 1e-06 | 2e-07 | ns |
|  | Stimuli - M2 (IL-4, IL-13) | -0.3(-0.4-0.3) | 0.3(0.1-0.3) | 0.3(0.2-0.4) | 8e-07 | 1e-06 | ns |
|  | Stimuli - M2 (IL-4) | -0.2(-0.3-0.1) | 0.2(0.2-0.3) | 0.2(0.2-0.3) | 6e-06 | 1e-04 | ns |
|  | Stimuli - M2 (IL-4, IL-13) | -0.2(-0.3-0.1) | 0.2(0.1-0.3) | 0.3(0.2-0.3) | 2e-07 | 5e-05 | ns |
|  | Stimuli - M2 (IL-4, IL-13) | -0.2(-0.3-0.1) | 0.2(0.1-0.3) | 0.1(0-0.2) | 1e-04 | 0.012 | ns |
|  | Stimuli - M2 (IL-4, IL-13) | 0.1(-0.1-0.2) | 0.1(0-0.2) | -0.1(-0.2-0) | ns | 0.035 | 6e-04 |
|  | Stimuli -Fatty acids (PA, SA) | -0.3(-0.4-0.1) | 0.3(0.2-0.4) | 0.2(0.1-0.3) | 4e-06 | 8e-06 | ns |
|  | Stimuli - Fatty acids (PA, SA) | -0.2(-0.4-0.1) | 0.3(0.2-0.3) | 0.2(0.1-0.3) | 4e-07 | 3e-06 | ns |
|  | Stimuli - Fatty acids (PA, LiA, OA) | -0.2(-0.2-0.1) | 0.2(0.1-0.2) | 0.2(0.1-0.3) | 4e-06 | 1e-04 | ns |
|  | Stimuli - Fatty acids (PA, SA, OA) | -0.3(-0.4-0.2) | 0.2(0.2-0.2) | 0.2(0.1-0.3) | 2e-07 | 4e-07 | ns |
|  | Stimuli - Fatty acids (PA, SA, LiA) | -0.1(-0.3-0) | 0.2(0.2-0.3) | 0.2(-0.1-0.4) | 7e-05 | 0.045 | ns |
|  | Stimuli - Fatty acids (PA, SA) | -0.1(-0.3-0.2) | 0.1(0.1-0.3) | 0(-0.1-0) | 0.004 | Ns | 9e-04 |
|  | Stimuli - Fatty acids (OA, LA, LiA) | -0.2(-0.3-0.1) | 0(0-0.3) | 0.1(0-0.3) | 6e-05 | 2e-05 | ns |
|  | Stimuli - Fatty acids (OA, LA, LiA) | -0.2(-0.3-0.1) | 0.3(0.2-0.3) | 0.3(0.2-0.3) | 2e-07 | 1e-04 | ns |
|  | Stimuli - Fatty acids (OA, LA, LiA) | -0.2(-0.3-0.2) | 0.3(0.2-0.3) | 0.3(0.2-0.4) | 2e-07 | 1e-05 | ns |
|  | Stimuli - Fatty acids (PA, LiA, SA) | -0.3(-0.4-0.1) | 0.3(0.3-0.4) | 0.3(0.3-0.4) | 2e-07 | 5e-05 | ns |
|  | Stimuli - Fatty acids (LiA, OA, PA) | -0.4(-0.5-0.3) | 0.4(0.3-0.4) | 0.4(0.3-0.4) | 2e-07 | 2e-07 | ns |
|  | Stimuli - Fatty acids (LA, LA, SA) | 0(0-0.1) | 0.3(0.1-0.4) | 0(-0.2-0.1) | 0.013 | Ns | 0.012 |
|  | Stimuli - Fatty acids (OA, LiA) | -0.4(-0.4-0.1) | 0.3(0.2-0.3) | 0.3(0.2-0.4) | 2e-06 | 3e-04 | ns |
|  | Stimuli - Others (TPP, TPP + IFN) | 0.2(0.1-0.2) | 0.2(0-0.3) | -0.2(-0.3-0.2) | ns | 1e-05 | 7e-06 |
|  | Stimuli - Others (TPP, TPP + IFN) | 0(-0.1-0.1) | 0.3(0.2-0.3) | 0.1(-0.1-0.2) | 9e-06 | Ns | 3e-04 |
|  | Stimuli - Fatty acids (PA, SA) | 0.1(-0.1-0.2) | 0.3(0.2-0.4) | 0(-0.1-0.1) | 4e-04 | Ns | 1e-04 |
|  | Stimuli - Others (TPP, TPP + IFN) | -0.1(-0.2-0) | 0.3(0.2-0.3) | 0.1(0-0.2) | 2e-06 | 0.005 | 0.003 |
|  | Stimuli - M1 (LPS) | -0.1(-0.2-0.1) | 0.2(0.1-0.3) | 0.1(0.1-0.3) | 4e-06 | 3e-04 | ns |
|  | Stimuli - Others (TPP, TPP + IFN) | -0.2(-0.3-0.1) | 0.3(0.2-0.4) | 0.3(0.2-0.4) | 2e-07 | 2e-04 | ns |
|  | Stimuli - M1 (IFN-γ + TNF-α) | -0.3(-0.4-0.1) | 0.4(0.3-0.4) | 0.3(0.2-0.3) | 2e-06 | 3e-04 | ns |
|  | Stimuli - Others (TPP) | -0.1(-0.1-0) | 0(-0.1-0.1) | 0.1(-0.1-0.2) | ns | Ns | ns |
|  | Stimuli - M2 (IL-4) | -0.3(-0.3-0.2) | 0.2(0.2-0.3) | 0.3(0.2-0.4) | 2e-07 | 1e-05 | ns |
|  | Stimuli - Others (TPP, TPP + IFN) | -0.2(-0.2-0) | 0.2(0.1-0.2) | 0.2(0-0.3) | 9e-06 | 5e-04 | ns |
|  | Stimuli - Others (TPP, TPP + IFN) | -0.2(-0.2-0.1) | 0.2(0.1-0.2) | 0.2(0.1-0.3) | 1e-06 | 6e-05 | ns |
|  | Stimuli - Others (TPP, TPP + IFN) | -0.1(-0.3-0) | 0.2(0.1-0.2) | 0.2(0.1-0.3) | 2e-05 | 3e-04 | ns |
|  | Stimuli - Others (GC) | -0.1(-0.2-0) | 0.2(0.1-0.3) | 0.1(0-0.3) | 8e-04 | 0.021 | ns |
|  | Stimuli - Others (GC) | -0.2(-0.4-0) | 0.2(0.1-0.2) | 0.3(0.1-0.4) | 2e-06 | 4e-05 | ns |
|  | Stimuli - Others (GC, HDL) | -0.1(-0.3-0) | 0.2(0.2-0.3) | 0.2(0.1-0.3) | 4e-06 | 3e-04 | ns |
|  | Stimuli - Fatty acids (OA) | -0.2(-0.3-0.1) | 0.2(0.2-0.3) | 0.2(0.1-0.3) | 2e-07 | 1e-04 | ns |
|  | Stimuli - M2 (IL-4, IL-10, IL-13) | -0.2(-0.3-0.1) | 0.2(0.2-0.3) | 0.2(0.1-0.3) | 2e-07 | 4e-04 | ns |
|  | Stimuli - M2 (IL-4, PGE2) | -0.2(-0.3-0.1) | 0.3(0.2-0.3) | 0.2(0.2-0.3) | 4e-07 | 1e-04 | ns |
|  | Stimuli - M2 (IL-4, PGE2) | 0(-0.2-0) | 0.2(0.1-0.2) | 0.2(0-0.3) | 0.005 | Ns | ns |
|  | Stimuli -M2 (IL-4, IL-13, PGE2) | -0.2(-0.2-0.1) | 0.2(0.1-0.2) | 0.2(0.1-0.3) | 4e-07 | 4e-06 | ns |
|  | Unstimulated | 0(-0.1-0.2) | 0.1(0-0.3) | 0.2(0.1-0.3) | ns | 0.026 | ns |
| **MyD88** | MyD88 cascade initiated on plasma membrane | 0.1(0-0.2) | 0.2(0.1-0.3) | 0.1(-0.2-0.2) | 0.039 | Ns | ns |
|  | MyD88 deficiency - TLR2/4 | 0.2(-0.2-0.2) | 0.3(0-0.4) | 0.2(-0.1-0.4) | ns | Ns | ns |
|  | MyD88 deficiency - TLR5 | 0.1(-0.2-0.6) | 0.3(-0.5-0.6) | 0.3(0-0.7) | ns | Ns | ns |
|  | MyD88 dependent cascade initiated on endosome | 0.1(0-0.2) | 0.3(0.1-0.3) | 0.1(-0.2-0.2) | 0.019 | Ns | ns |
|  | MyD88 independent TLR4 cascade | 0(-0.1-0.2) | 0.2(0.2-0.3) | 0.1(-0.2-0.3) | 0.012 | Ns | 0.050 |
|  | MyD88 MAL TIRAP cascade initiated on plasma membrane | 0.1(-0.1-0.2) | 0.3(0.1-0.3) | 0.1(-0.2-0.3) | 0.034 | Ns | 0.032 |

**Supplementary Table S11- Distribution of individuals with mild-moderate asthma (MMA) and severe (SA) asthma among clusters (OACs).**

|  | **MMA** | **SA** |
| --- | --- | --- |
| **OAC1** | 11 | 9 |
| **OAC2** | 1 | 12 |
| **OAC3** | 0 | 12 |
| **OAC4** | 0 | 13 |
| **OAC5** | 3 | 11 |


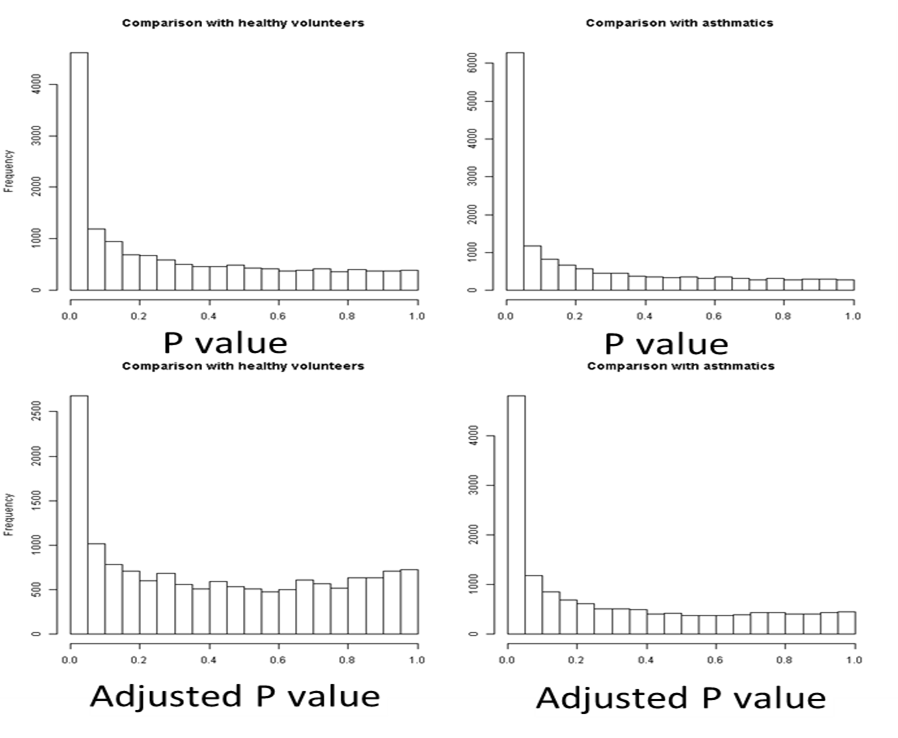


**Supplementary Figure S1**. Histogram of P values and adjusted P values by FDR (Q values). The top two histograms shows the distribution of P value for all group comparisons across healthy and OACs (on the left), and intra OACs (on the right).The bottom two histograms shows distributions of the P values after adjustment by FDR.

**
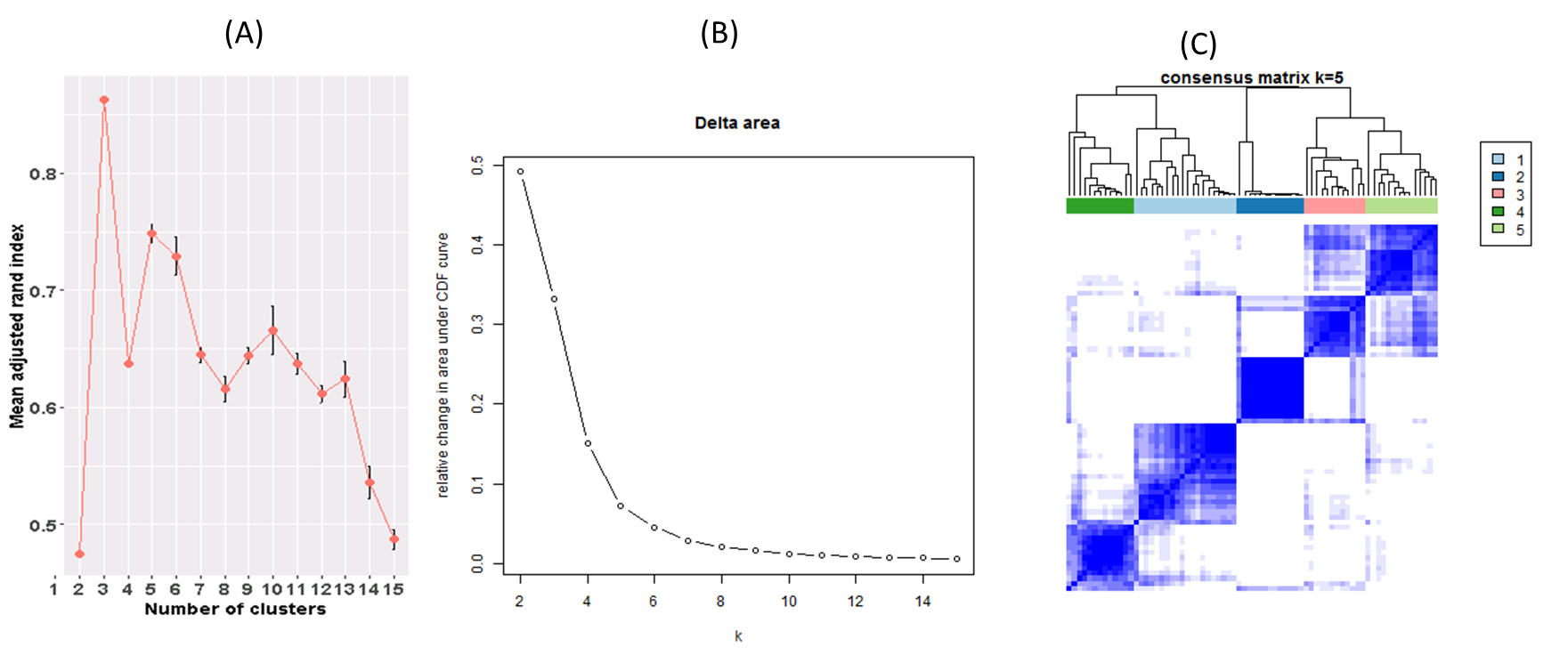
**

**Supplementary Figure S2: Between methods agreement and** Consensus clustering to determine optimal number of clusters. A) Five clusters shows the highest adjusted rand index after that for three clusters. It also has a small variance. This shows that this clustering result is less sensitive to clustering methods over a wide variation of combination of the data. Therefore, might be most stable clustering result among the 13*2*7 combinations. (C) CDF: cumulative distribution function. The optimal cluster number was determined by finding a cluster number k that exhibits a relative small increase of the relative change of the CDF at the k+1 cluster the increase of relative amount of change after k=5 was minimal and stable. Cluster number k=5 was the optimal choice.

**Supplementary Figure S3:** Greater gene expression (log2 intensity) of T2 cytokines and other mediators in OAC3 than other OACs.

BCL2 mRNA (log intensity)


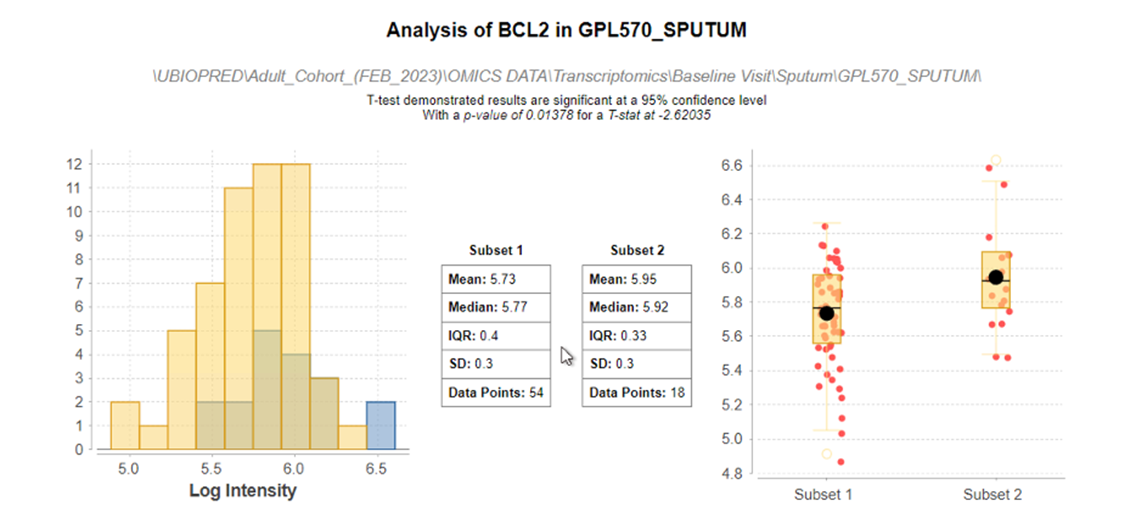

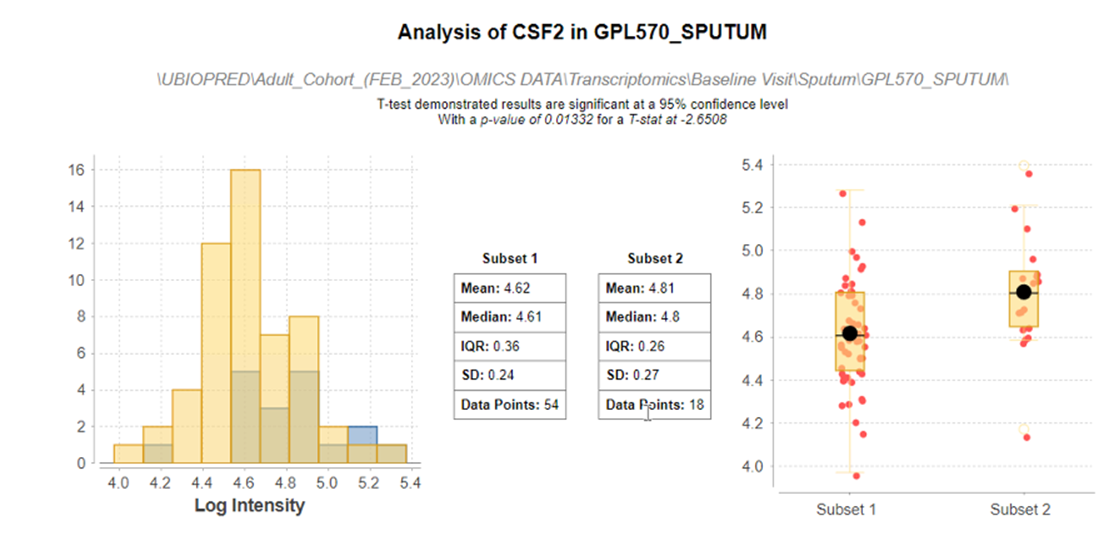


CSF-2 mRNA (log intensity)

A

B

C

D

E

F


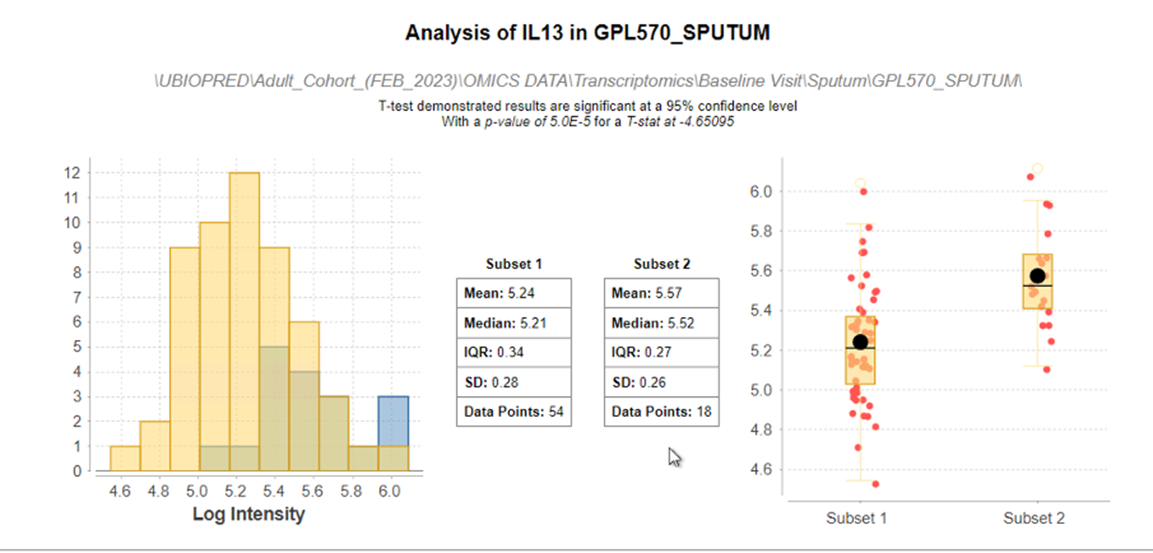


IL-13 mRNA (log intensity)

OAC3

Non-OAC3

p=5x10^-5^

p=0.01378

p=0.01332


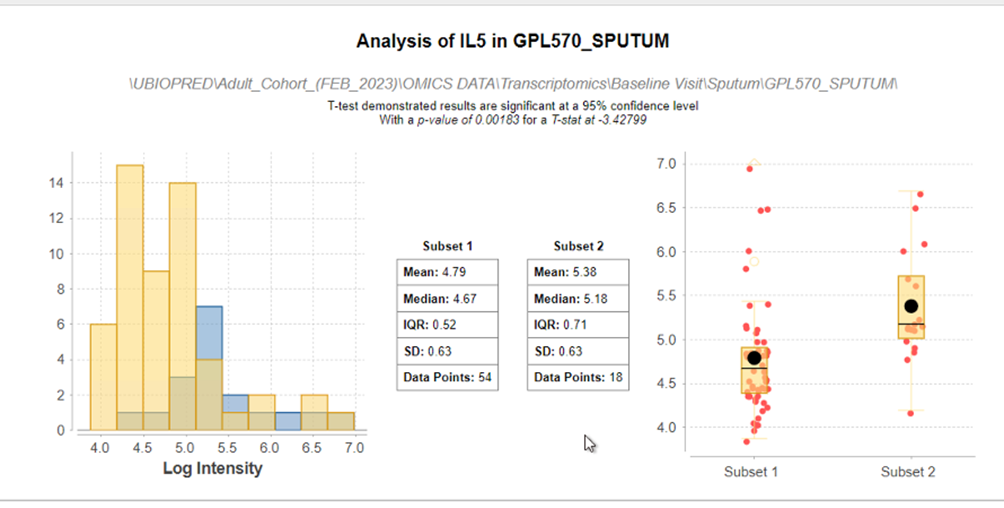


IL-5 mRNA (log intensity)

p=0.00183

OAC3

Non-OAC3


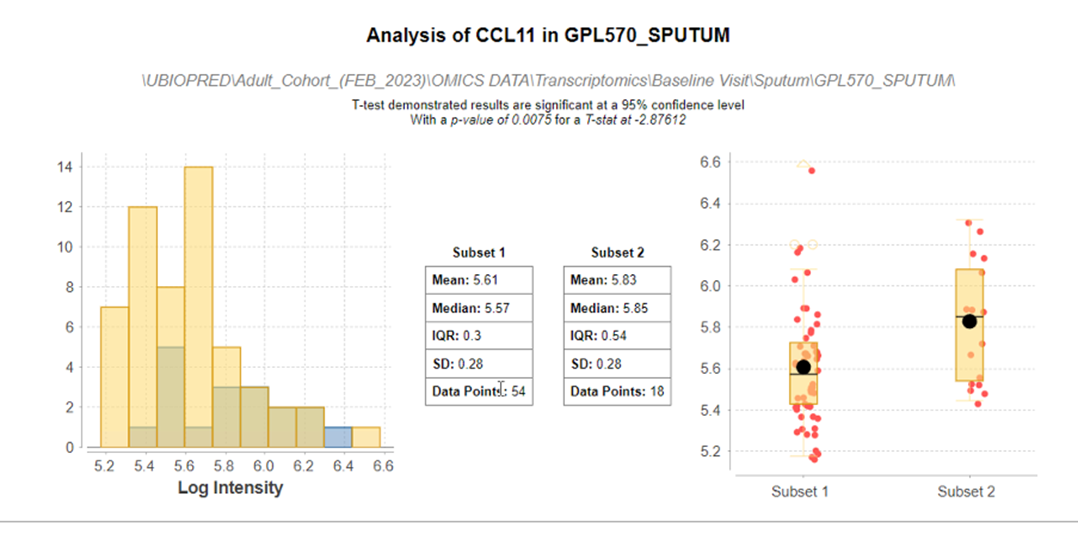


CCL11 mRNA (log intensity)

p=0.0075

OAC3

Non-OAC3


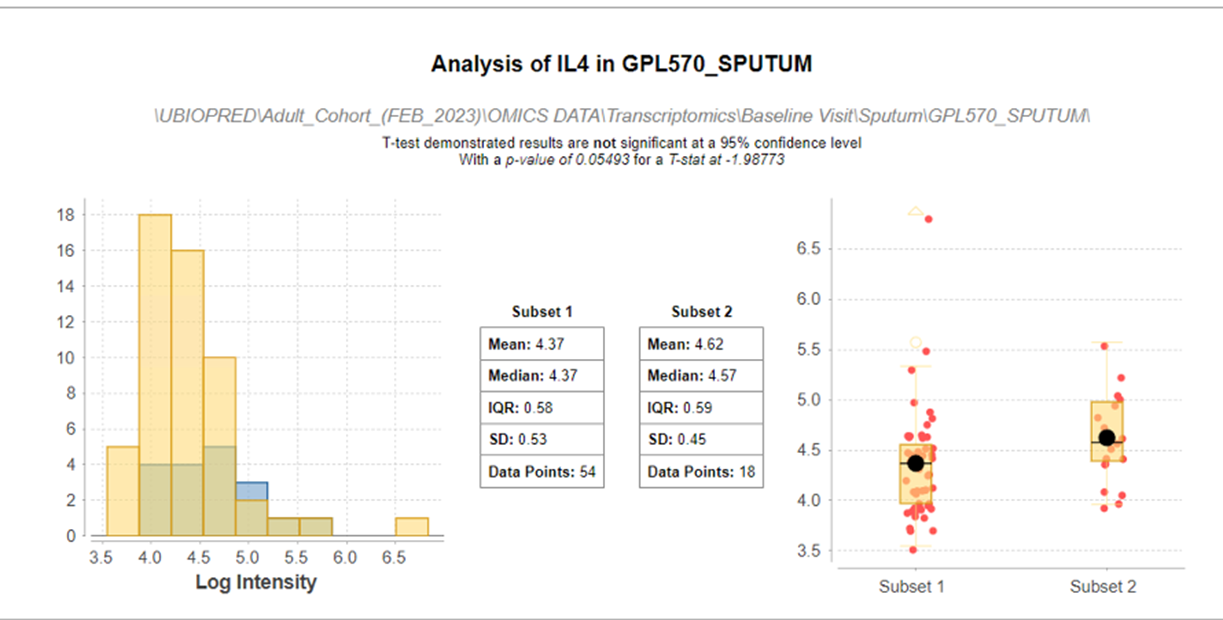


IL-4 mRNA (log intensity)

p=0.05493

OAC3

Non-OAC3

OAC3

Non-OAC3

OAC3

Non-OAC3


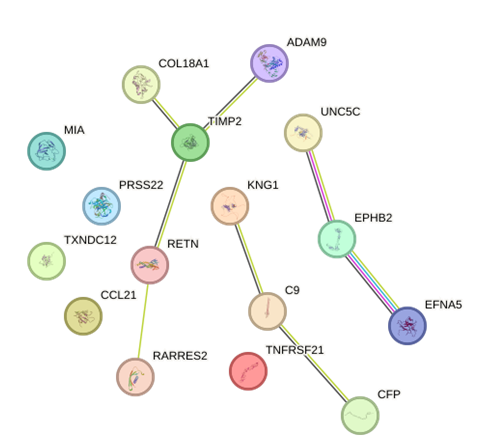


**Supplementary Figure S4-** Visualization of the protein-protein interaction network generated by STRING-DB (version 12.0), illustrating interactions among proteins enriched in SA compared to MMA within OAC1, after adjustment for clinical differences. The network depicts relationships between proteins based on experimental and predicted interaction data, providing insights into potential molecular mechanisms underlying severe asthma pathogenesis.

**
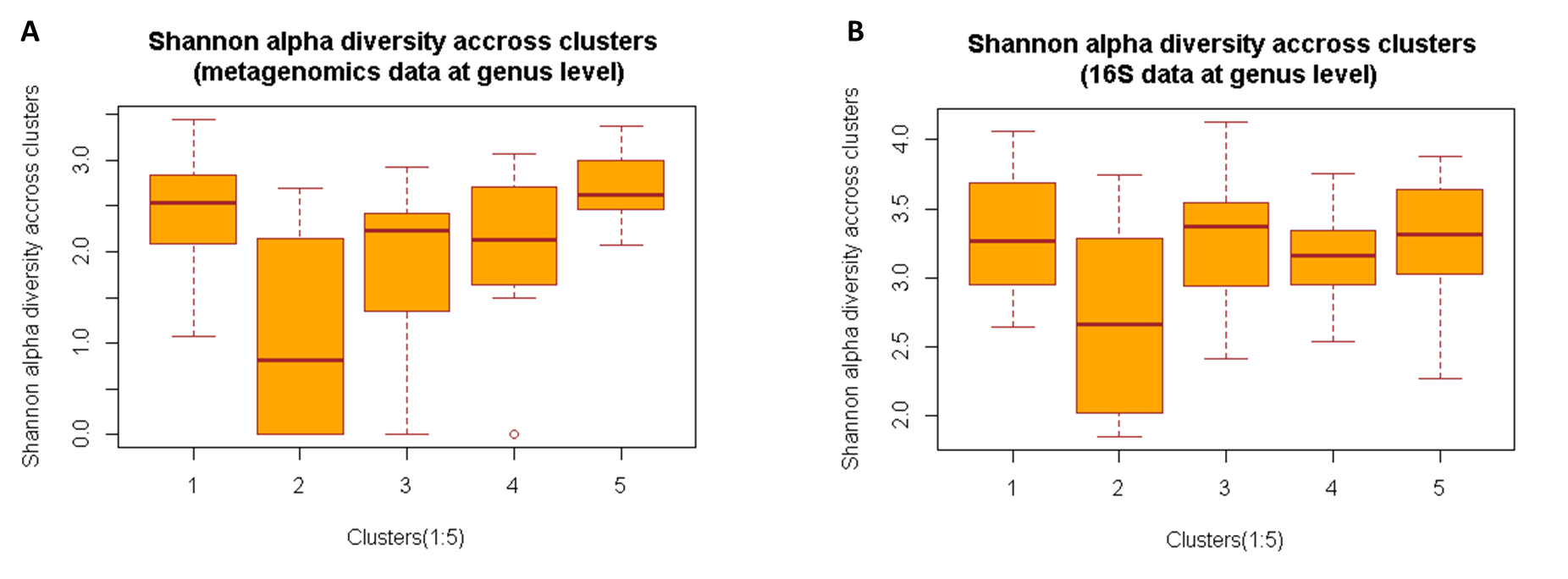
**

**Supplementary Figure S5:** Reduced microbial diversity (Shannon index) in OAC2 compared to other OACs.

**
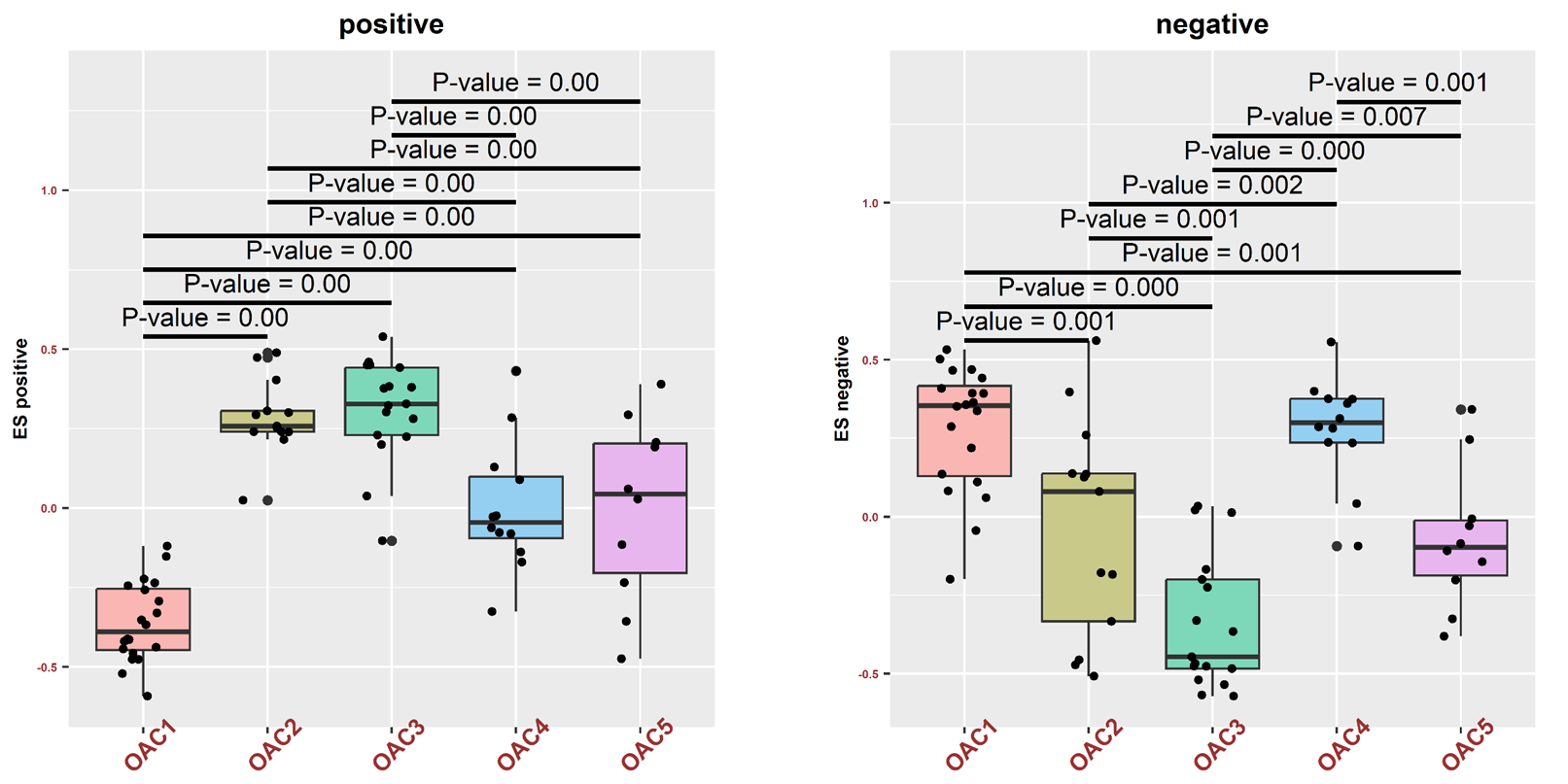
**

**Supplementary Figure S6.**  Enrichment scores (ES) of ‘Eosinophil survival in Asthma’ designed by associations with eosinophil numbers in asthma.

**
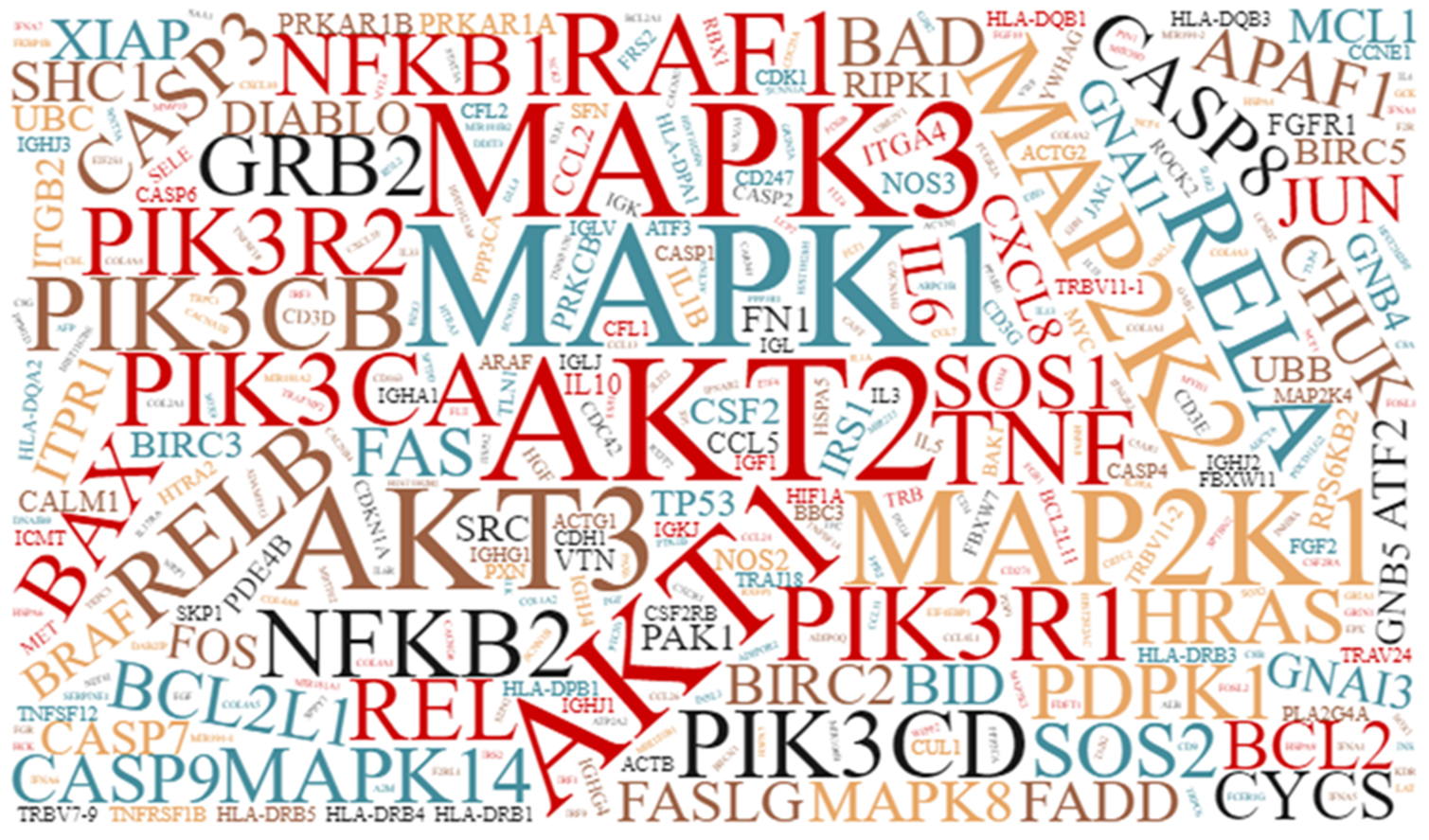
**

**Supplementary Figure S7:** Word cloud visualization of differentially activated pathways across the 5 omics-associated clusters (OACs). The figure is generated using https://www.wordclouds.com/.genes belonging to pathways that consistently perturbed across OACs.

**
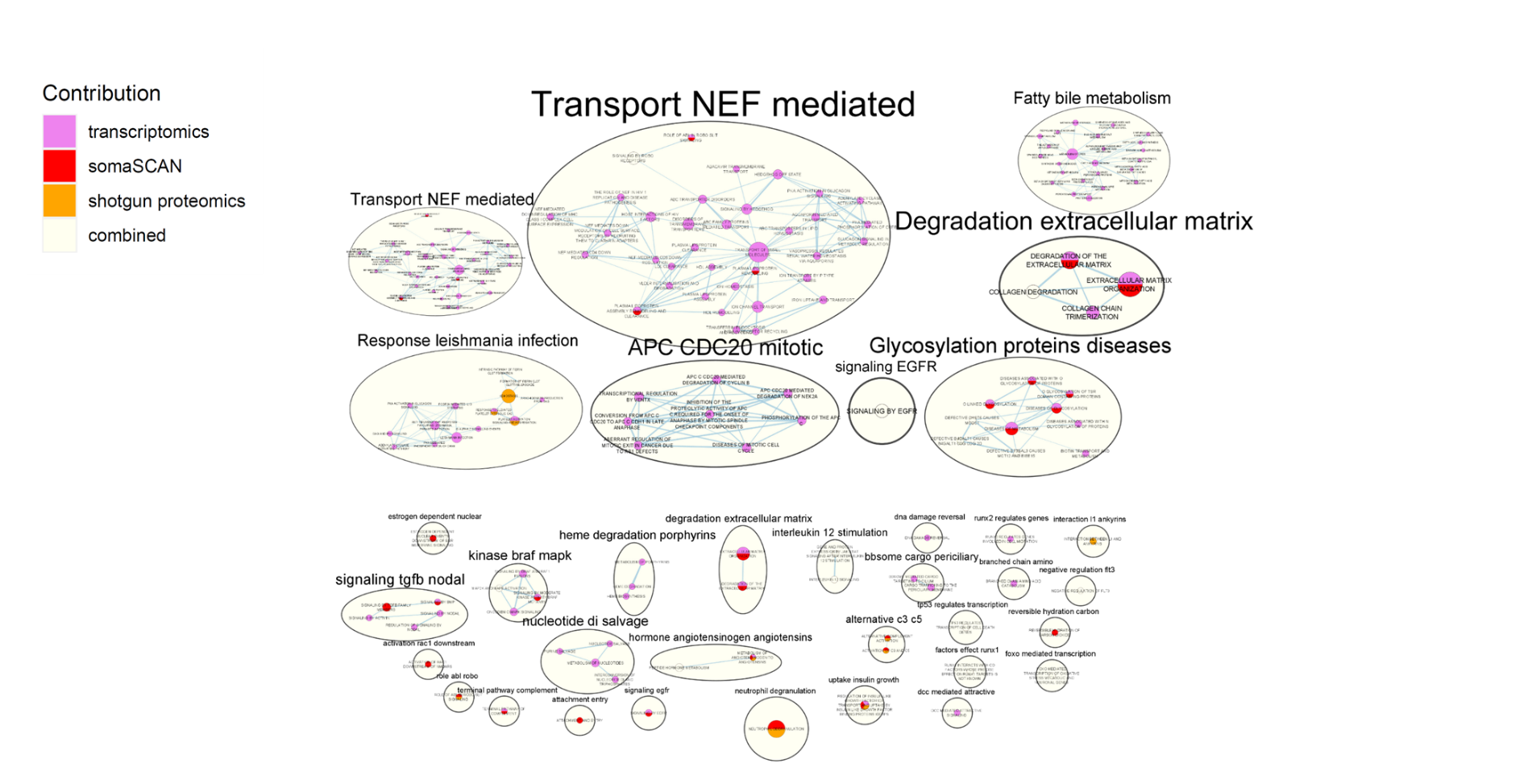
**

**Supplementary Figure S8:** Active multiomic pathway analysis of OAC1

**
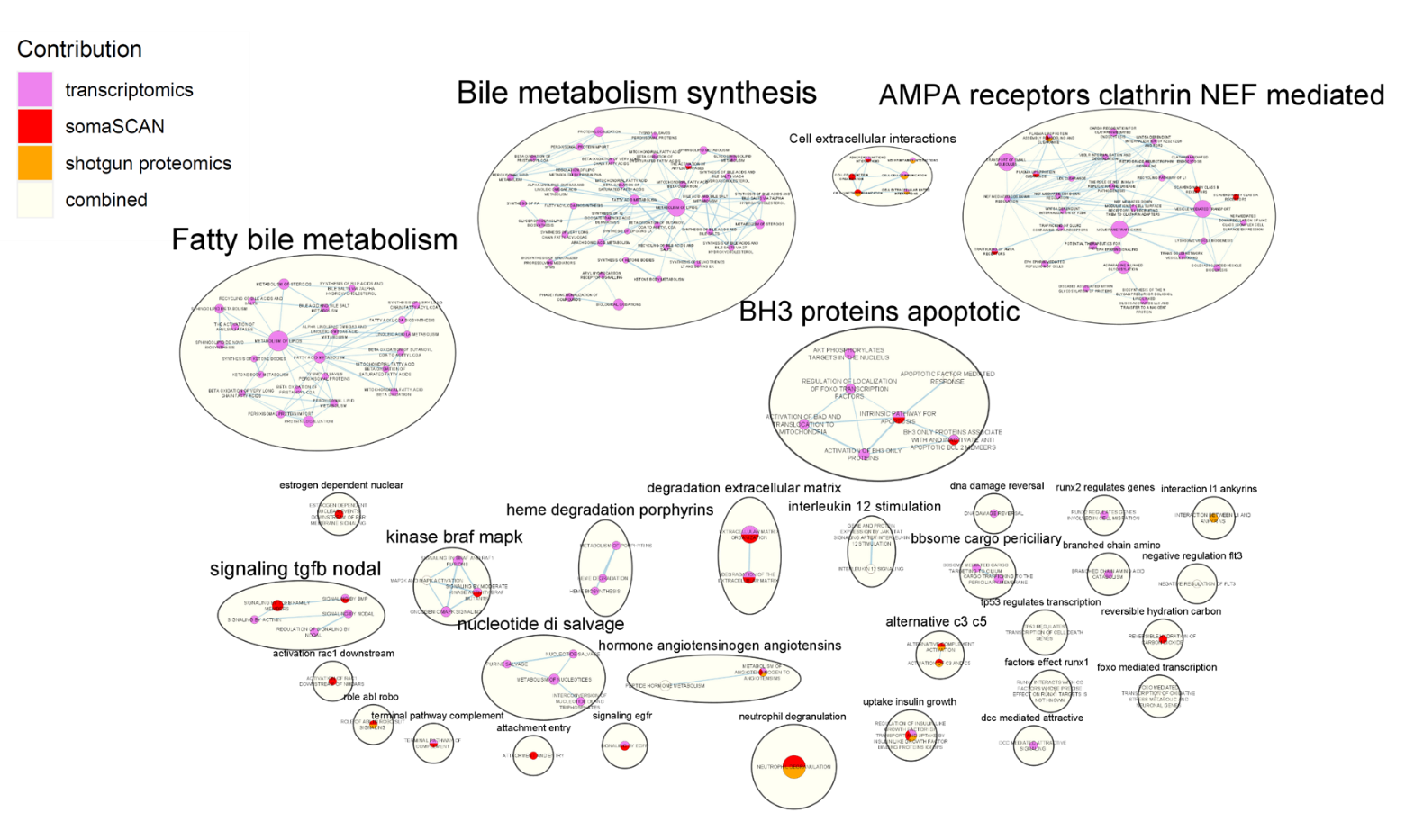
**

**Supplementary Figure S9:** Active multiomic pathway analysis of OAC2

**
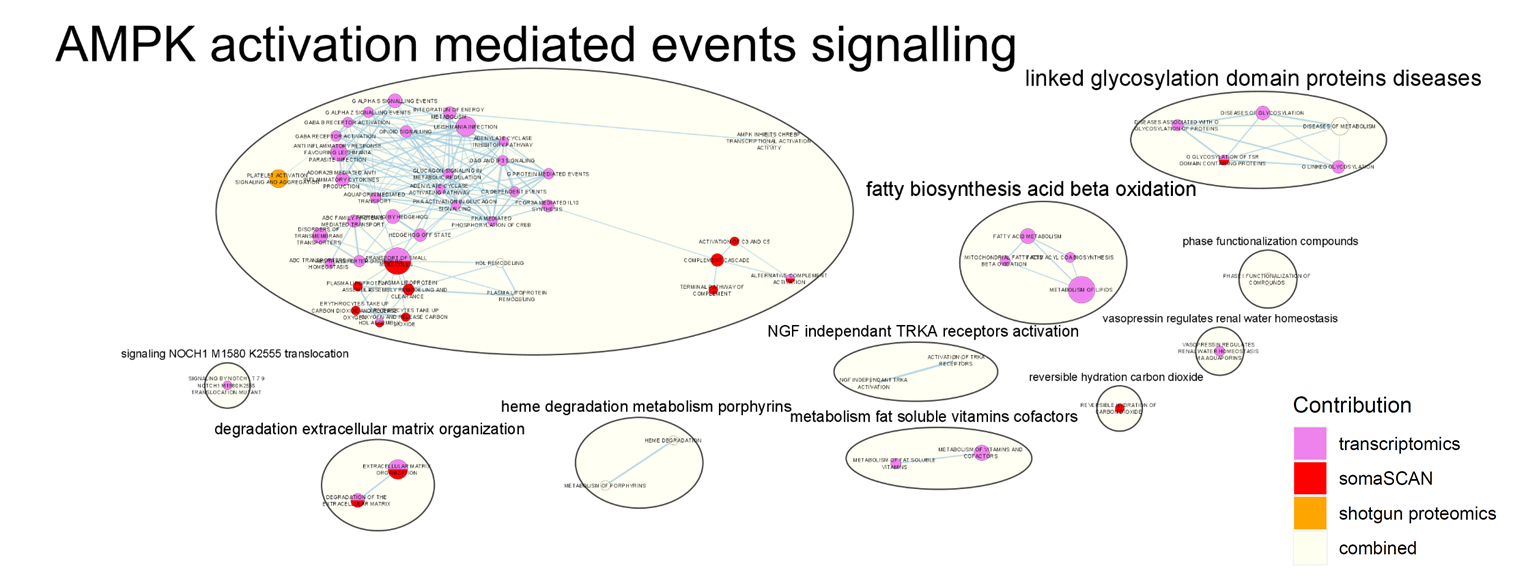
**

**Supplementary Figure S10:** Active multiomic pathway analysis of OAC3

**
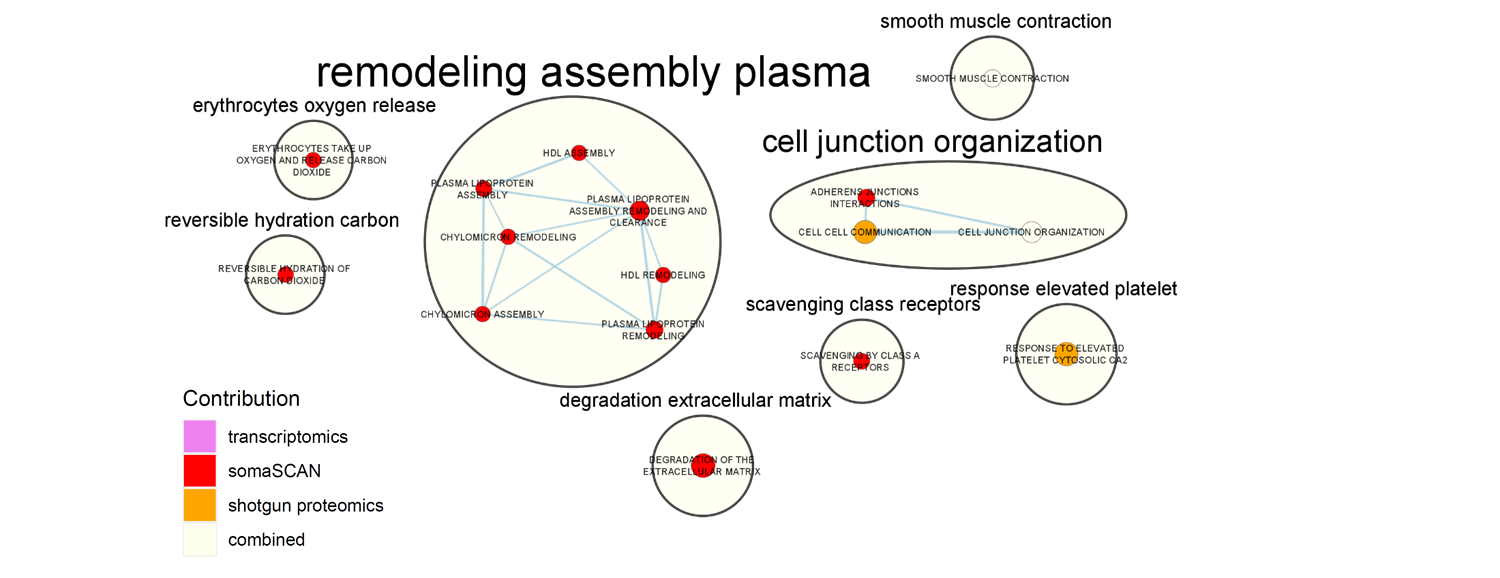
**

**Supplementary Figure S11:** Active multiomic pathway analysis of OAC5

**
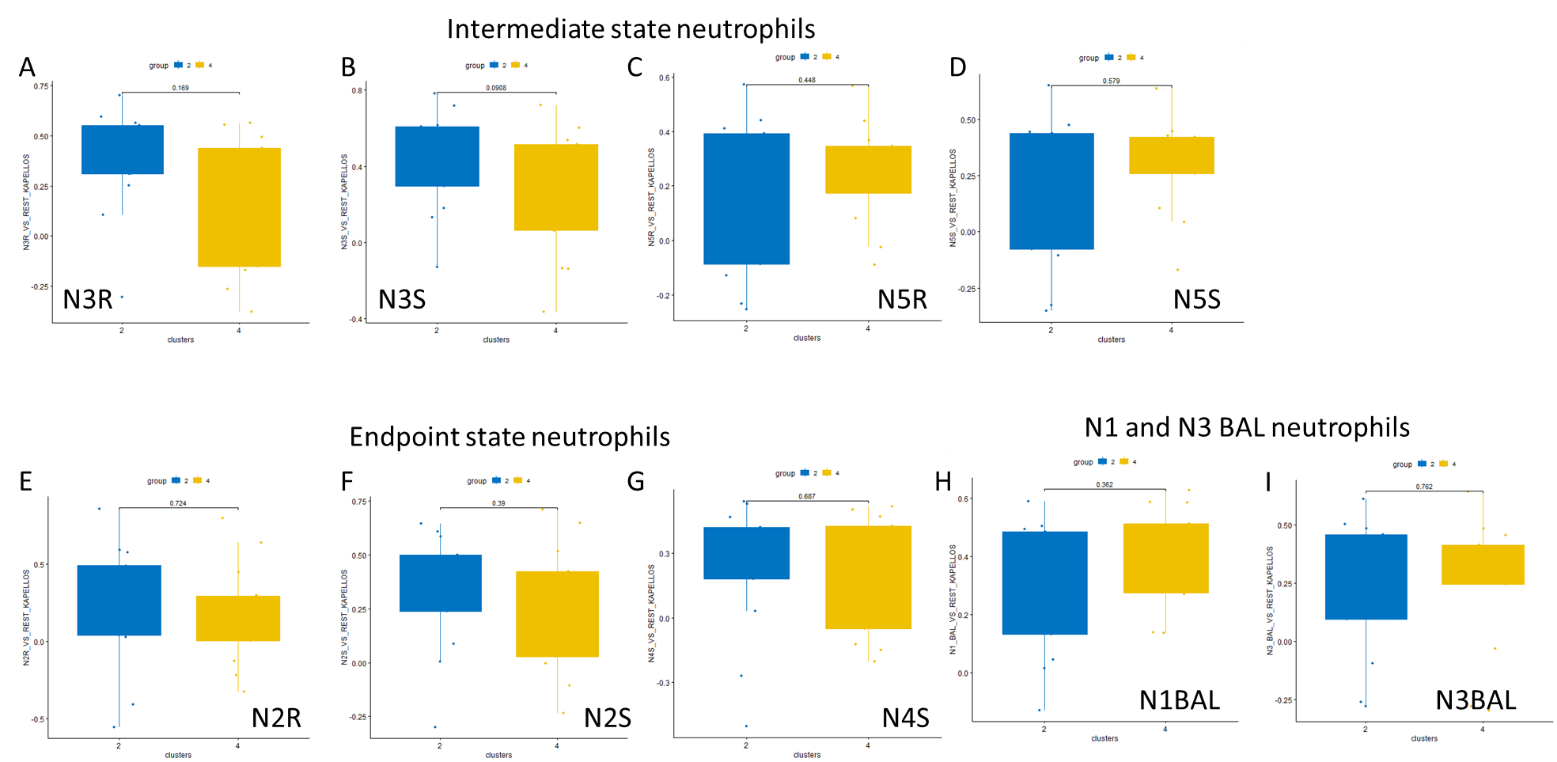
**

**Supplementary Figure S12.** Enrichment scores (ES) of neutrophil signatures in OAC2 compared with OAC4 using GSVA.
